# Pertussis toxin suppresses dendritic cell-mediated delivery of *B. pertussis* into lung-draining lymph nodes

**DOI:** 10.1371/journal.ppat.1010577

**Published:** 2022-06-06

**Authors:** Nela Klimova, Jana Holubova, Gaia Streparola, Jakub Tomala, Ludmila Brazdilova, Ondrej Stanek, Ladislav Bumba, Peter Sebo

**Affiliations:** 1 Institute of Microbiology of the Czech Academy of Sciences,Prague, Czech Republic; 2 Faculty of Sciences, Charles University, Prague, Czech Republic; 3 Czech Centre for Phenogenomics BIOCEV, Vestec, Czech Republic; 4 Institute of Biotechnology of the Czech Academy of Sciences, Vestec, Czech Republic; University of California Davis School of Medicine, UNITED STATES

## Abstract

The adenylate cyclase (ACT) and the pertussis (PT) toxins of *Bordetella pertussis* exert potent immunomodulatory activities that synergize to suppress host defense in the course of whooping cough pathogenesis. We compared the mouse lung infection capacities of *B*. *pertussis* (*Bp*) mutants (*Bp* AC^−^ or *Bp* PT^–^) producing enzymatically inactive toxoids and confirm that ACT action is required for maximal bacterial proliferation in the first days of infection, whereas PT action is crucial for persistence of *B*. *pertussis* in mouse lungs. Despite accelerated and near complete clearance from the lungs by day 14 of infection, the PT^−^ bacteria accumulated within the lymphoid tissue of lung-draining mediastinal lymph nodes (mLNs). In contrast, the wild type or AC^−^ bacteria colonized the lungs but did not enter into mLNs. Lung infection by the PT^−^ mutant triggered an early arrival of migratory conventional dendritic cells with associated bacteria into mLNs, where the PT^−^ bacteria entered the T cell-rich paracortex of mLNs by day 5 and proliferated in clusters within the B-cell zone (cortex) of mLNs by day 14, being eventually phagocytosed by infiltrating neutrophils. Finally, only infection by the PT^−^ bacteria triggered an early production of anti-*B*. *pertussis* serum IgG antibodies already within 14 days of infection. These results reveal that action of the pertussis toxin blocks DC-mediated delivery of *B*. *pertussis* bacteria into mLNs and prevents bacterial colonization of mLNs, thus hampering early adaptive immune response to *B*. *pertussis* infection.

## Author summary

Of the three classical *Bordetella* species causing respiratory infections in mammals, only the human-specialized whooping cough agent *B*. *pertussis* produces the pertussis toxin (PT) as its major virulence factor. Human pertussis is an acute respiratory illness and the pleiotropic activities of pertussis toxin account for the characteristic systemic manifestations of the disease, such as hyperleukocytosis, histamine sensitization, hyperinsulinemia, or inflammatory lung pathology. We found that PT activity inhibits the migration of infected dendritic cells from the lungs into the draining mediastinal lymph nodes (mLNs). This prevents mLN infection by bacteria evading from migratory cells and delivery of bacterial antigens into mLNs. As a result, the induction of adaptive serum antibody responses to infection is delayed. We thus propose that PT action serves to create a time window for proliferation of *B*. *pertussis* on airway mucosa to facilitate transmission of the pathogen among humans.

## Introduction

Pertussis, or whooping cough, is an acute respiratory illness caused by *Bordetella pertussis* (*Bp*) that used to be the leading cause of infant mortality in the pre-vaccine era [1–3]. Despite global vaccine coverage, pertussis burden remains high and it is estimated that ~24 million whooping cough cases and ~160,000 pertussis-related deaths occur annually world-wide [4]. Moreover, pertussis is on the rise in developed countries using the acellular pertussis (aP) vaccines that confer a short-lasting protection and fail to prevent *B*. *pertussis* transmission by vaccinated individuals [5,6]. Indeed, data from animal models show that the aP vaccine does not prevent nasopharynx infection by *B*. *pertussis* [7–12]. *B*. *pertussis* attaches to ciliated epithelia of the airways by means of several adhesins, evades the first line of host innate defense by deploying several complement resistance factors and subverts host immunity by the synergy of pertussis toxin (PT) and adenylate cyclase toxin-hemolysin (ACT, AC-Hly or CyaA) activities [3]. PT and ACT deliver their cytotoxic enzyme subunits into an array of immune cells and blunt the bactericidal functions of phagocytes and hamper induction of adaptive immune responses through hijacking of cellular signaling pathways [13–16].

ACT is produced also by *B*. *parapertussis* and *B*. *bronchiseptica* and was proposed to play an important role in the early stages of airway colonization [17–20]. The 177 kDa-large ACT protein harbors an N-terminal adenylyl cyclase (AC) enzyme domain (~40 residues) that is fused to a ~1,306 residue-long RTX hemolysin (Hly) moiety [21]. Hly binds the complement receptor 3 (CR3, α_M_β_2_ integrin CD11b/CD18, or Mac-1) of myeloid phagocytes [22–26], penetrates phagocyte membrane and delivers into cells the AC domain [27]. The AC enzyme is activated by cytosolic calmodulin and catalyzes a rapid and unregulated conversion of cellular ATP into the key second messenger molecule cAMP [28–30]. Increased cAMP levels deregulate cellular signaling pathways and ablate key innate immunity mechanisms, such as the oxidative burst of neutrophils [31–34], bactericidal NO production of macrophages [35] and the opsonophagocytic capacities of myeloid phagocytes [36–40]. The ACT-produced cAMP signaling also blocks transition of incoming monocytes into the more bactericidal macrophages and triggers dedifferentiation and apoptosis of tissue-resident macrophages [13,14,41–44]. Finally, ACT-produced cAMP elevation hampers also the adaptive immune responses, inhibiting dendritic cell (DC) maturation and blocking antigen presentation to T cells [45,46].

In contrast to ACT, PT is only produced by *B*. *pertussis* and represents its major virulence factor, exerting both local and systemic immunomodulatory effects [16]. Systemic effects of PT action account for the characteristic manifestations of pertussis, such as hyperleukocytosis due to leukocyte proliferation, lymphocyte egress from lymphoid organs and bone marrow into circulation and inhibition of leukocyte extravasation [47]. PT action leads to formation of mixed lymphocyte aggregates in arterioles, triggering pulmonary hypertension and eventual heart failure [48,49], as well as histamine sensitization [50]. Moreover, PT action plays a key role in immune evasion of the bacterium, delaying neutrophil recruitment onto the infected mucosa and interfering with induction of adaptive immune responses [17,51–55].

PT is formed by six subunits (S1:S2:S3:2S4:S5) that assemble into a typical AB_5_ toxin oligomer, with an ADP-ribosyl transferase enzyme subunit A (S1) capping a pentameric B_5_ subunit. The B_5_ subunit mediates toxin binding to surface sialoglycoproteins and itself triggers mitogenic signaling and DC maturation [56–59]. PT enters cells by receptor-mediated endocytosis and upon retrograde transport reaches the endoplasmic reticulum, where the enzymatic A subunit dissociates and translocates into the cell cytosol [60]. Using cytosolic NAD as a substrate, the A subunit ADP-ribosylates and inactivates the inhibitory G_i/o_α subunits of heterotrimeric G proteins, thereby disrupting the inhibitory GPCR signaling and relieving the inhibition of endogenous adenylyl cyclase enzymes by G_i/o_α [60–62]. The resulting increase of cellular cAMP levels dysregulates protein kinase A-activated signaling pathways depending on various GPCRs and the activities of Ca^2+^ and K^+^ channels in various cell types [63,64]. Since most chemokine receptors are coupled to G_i/o_ proteins [65], one action of PT is to deregulate the chemotaxis of macrophages, lymphocytes and neutrophils [51,52,66–68].

Previously, PT activity was found to reduce CCR7-mediated *ex vivo* migration of human monocyte-derived dendritic cells (MDDCs) towards the lymphoid chemokine CCL21 [69]. Moreover, an enhanced migration of CD11c^+^ cells from mouse lungs into the lung-draining mediastinal lymph nodes (mLNs) was observed upon lung infection with a *B*. *pertussis* mutant lacking genes for PT production [67]. On the other hand, we have previously observed that the cAMP-elevating activity of ACT enhanced migration of TLR-activated mouse and human DC towards lymph node-homing chemokines CCL19 and CCL21 [46]. Therefore, we tested here the hypothesis that PT activity delays ACT-triggered migration of *B*. *pertussis* antigen*-*loaded DCs from infected lungs into mLNs and show that early DC-mediated delivery of PT-deficient bacteria enables *B*. *pertussis* proliferation in mLNs.

## Results

### Pertussis toxin prevents prolonged *B*. *pertussis* infection of lung-draining lymph nodes

**Fig 1:**
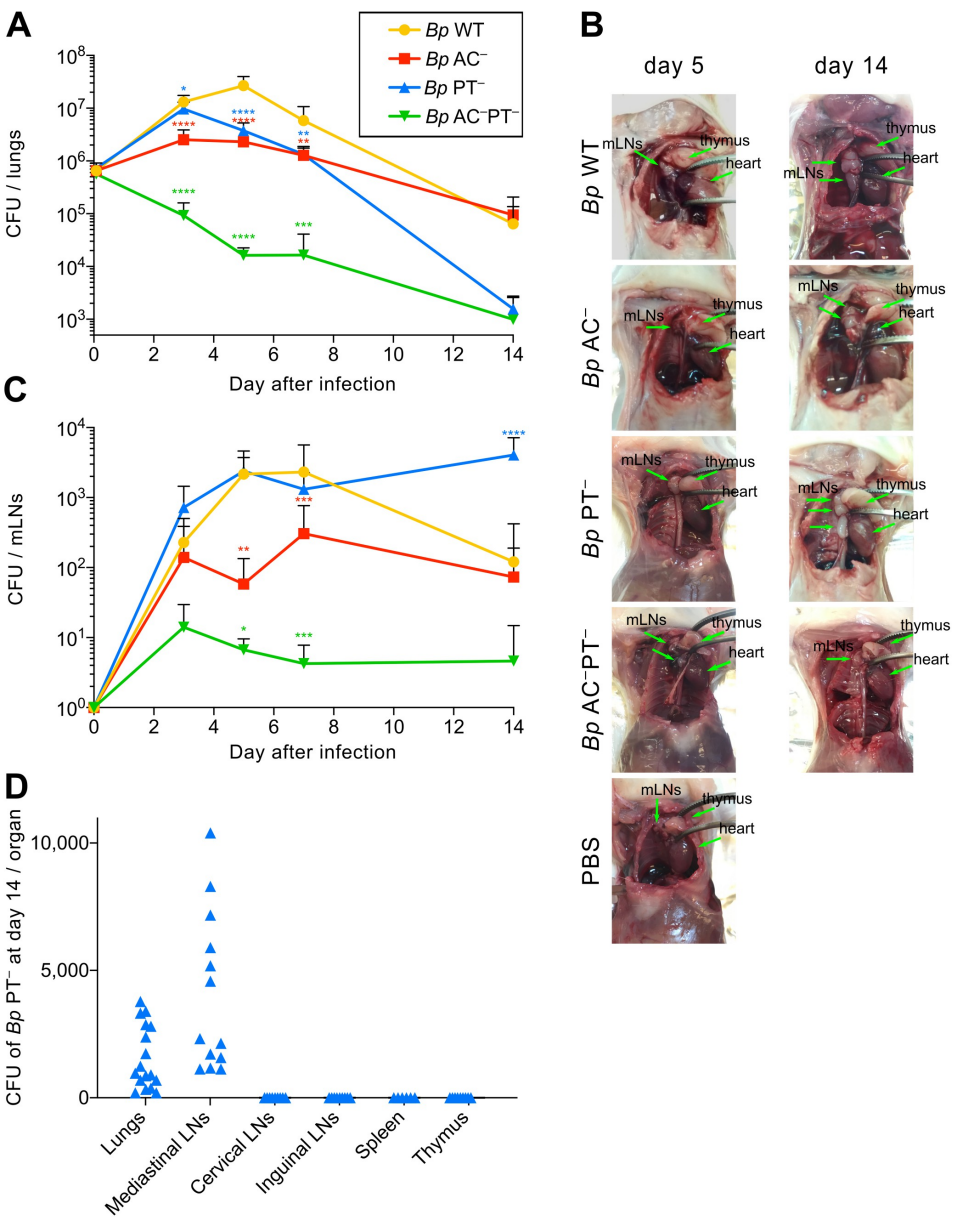
PT-deficient *B*. *pertussis* is cleared from lungs but persists in mediastinal lymph nodes. **(A)** *B*. *pertussis* colonization of lungs after intranasal administration of the wild-type (*Bp* WT) and mutant *B*. *pertussis* strains producing the catalytically-inactive pertussis toxin (PT^–^), the catalytically-inactive adenylate cyclase toxin (AC^–^) or a combination of both toxoids (AC^–^PT^–^). Four-week-old BALB/c mice were intranasally challenged with 8 × 10^5^ CFU (in 50 μl) of the bacteria expressing the mScarlet fluorescent protein. The total number of bacteria in the lungs at the indicated time points was determined by CFU counting upon plating of lung homogenates on BG blood agar plates. Data represent the mean values with standard deviation obtained from groups of at least three mice per time point in three independent experiments. Two-way ANOVA followed by Dunnett’s multiple comparisons test was used to analyze statistical significance between groups. * (p < 0.05), ** (p < 0.01), *** p (< 0.001), **** (p < 0.0001). **(B)** Examination of mediastinal lymph nodes (mLNs) on day 5 and 14 post infection. The location of the mLNs, thymus, and heart is indicated by arrows. **(C)** *B*. *pertussis* colonization of mLNs. The mLNs of a mouse sacrificed at the indicated time point were pooled and the homogenate was plated on BG blood agar for subsequent CFU counting. Data represent the means with standard deviations obtained from at least three mice per group and time point in three independent experiments. Two-way ANOVA followed by Dunnett’s multiple comparisons test was used to analyze statistical significance between groups. * (p < 0.05), ** (p < 0.01), *** p (< 0.001), **** (p < 0.0001). **(D)** *B*. *pertussis* colonization of lungs and lymphoid organs on day 14 after intranasal administration of the *B*. *pertussis* PT^−^ strain. The number of bacteria in the lungs and lymphoid organs on day 14 postinfection was determined by plating of organ homogenates on BG blood agar. LNs from each mouse were pooled.

To dissect the impact of ACT and PT activities on outmigration of DCs from *B*. *pertussis*-infected lungs into mLNs, we used Tohama I-derived mutants (Table 1) producing enzymatically inactive toxoids of ACT or PT (*Bp* AC^−^ or *Bp* PT^–^) [44]. The used bacteria further produced the fluorescent mScarlet protein from a plasmid [70]. This did not affect growth or secretion of the AC^−^ and PT^−^ toxoids by the bacteria *in vitro* and enabled *B*. *pertussis* imaging in the tissues of infected mice for up to 14 days without the need for antibiotic selection (S1 Fig and below). In line with intact fitness *in vitro*, the *Bp* WT bacteria (AC^+^PT^+^) inoculated intranasally (8 x 10^5^ CFU in 50 μL) proliferated in the lungs of adult BALB/c mice by ~1.5 order of magnitude within 5 days (to ~2.7 x 10^7^ CFU) and persisted after a progressive decline at ~10^5^ CFU/lung on day 14 (Fig 1A). Under the same conditions, the *Bp* AC^−^ bacteria producing the ACT toxoid and active PT (AC^–^PT^+^) proliferated less and reached an order of magnitude lower CFU level in the lungs, while reproducibly persisting at a comparably high (~10^5^ CFU/lung) level as *Bp* WT by day 14. In contrast, the *Bp* PT^−^ bacteria, secreting the PT toxoid and producing active ACT (AC^+^PT^–^), proliferated as rapidly as *Bp* WT bacteria for the first 3 days of infection, but their CFU counts in the lungs rapidly decreased thereafter to only ~10^3^ CFU/lung on day 14 (Fig 1A). These results corroborate the previous conclusion, reached under different experimental conditions, that ACT plays an important role in the early phases of *B*. *pertussis* infection, whereas PT activity is important for sustained lung infection [17,19,20,55,67,71]. In fact, we have repeatedly found that the PT-producing *Bp* AC^−^ bacteria tended to persist in mouse lungs at detectable levels weeks after the wild-type or PT^−^ bacteria have already been cleared (S2 Fig and [71]).

**Table 1:** *B*. *pertussis* strains used in this study.

Strain (*)	Adenylate cyclase toxin (ACT)	Pertussis toxin (PT)
***Bp* WT** (*B*. *pertussis* parental strain)	active	active
***Bp* AC** ^–^	enzymatically inactive ACT (D_188_GSI_189_)	active
***Bp* PT** ^–^	active	enzymatically inactive PT subunit S1 (R9K E129G)
***Bp* AC**^–^ **PT**^–^	enzymatically inactive ACT (D_188_GSI_189_)	enzymatically inactive PT subunit S1 (R9K E129G)

*The *Bp* AC^–^ and *Bp* AC^–^PT^–^ strains secrete an enzymatically inactive adenylate cyclase toxoid (AC^–^) due to disruption of the catalytic site of the adenylyl cyclase enzyme by a GlySer dipeptide inserted between residues 188 and 189 [104]. The *Bp* PT^–^ and *Bp* AC^–^PT^–^ strains secrete the genetically detoxified PT toxoid (PT^–^) having the ADP-ribosylating enzyme activity disrupted by the R9K and E129G substitutions in the enzymatic S1 subunit of the pertussis toxin (or R43K and E163G, respectively, because this traditional residue numbering disregards the presence of processed signal peptide of the S1 subunit for historical reasons) [105].

Intriguingly, at a lower level of lung damage (S3 Fig) and at a comparable bacterial load in the lungs (*cf*. Fig 1A), the animals infected by the *Bp* PT^−^ bacteria had strikingly larger lung-draining lymph nodes on day 5 than the animals infected by the *Bp* AC^−^ strain (Fig 1B). The size of mLNs of *Bp* PT^–^-infected mice was comparable to that of animals infected by the *Bp* WT strain that by day 5 of infection proliferated to an order of magnitude higher CFU level in mouse lungs (*cf*. Fig 1A). Moreover, the difference of mLN size between animals infected by the *Bp* PT^−^ and *Bp* AC^−^ strains persisted through to day 14 (Fig 1B), even though the CFU counts of the *Bp* AC^−^ bacteria in the lungs remained two orders of magnitude higher than those of the *Bp* PT^−^ bacteria (*cf*. Fig 1A). Unexpectedly, an opposite trend was reproducibly observed when viable bacteria present in the mLNs were enumerated by plating of mLN homogenates (Figs 1C and S4). The ACT-producing *Bp* PT^−^ bacteria (AC^+^PT^–^) reached the mLNs at higher numbers than *Bp* WT (AC^+^PT^+^) already on day 3 of infection and persisted at numbers increased to ~4 x 10^3^ CFUs in the mLNs on day 14 of infection, thus exceeding the *Bp* PT^−^ CFU counts in the lungs (*cf*. Fig 1A). In contrast, the number of viable *Bp* WT bacteria in the mLNs mirrored their CFU counts in the lungs, peaking at ~10^3^ CFUs on day 7 and decreasing to ~10^2^ CFUs in mLNs by day 14. Moreover, the *Bp* AC^−^ (AC^–^PT^+^) bacteria were detected in the mLNs only at an order of magnitude lower level of ~10^2^ CFU over the 14 days of infection, despite persisting in the lungs (*cf*. Fig 1A). Finally, the *Bp* AC^–^PT^−^ bacteria producing the combination of the two toxoids were unable to proliferate in the lungs and were found in mLNs at very low numbers (~10 CFU) (Fig 1A and 1C). Hence, in the absence of PT activity, the production of active ACT favored early arrival of the *Bp* PT^−^ mutant into mLNs, where the bacteria persisted at increased numbers by day 14, but did not disseminate further into spleen or other lymphatic tissue (Fig 1D).

### PT-deficient *B*. *pertussis* invades the lymphoid tissue and proliferates in mLNs

**Fig 2:**
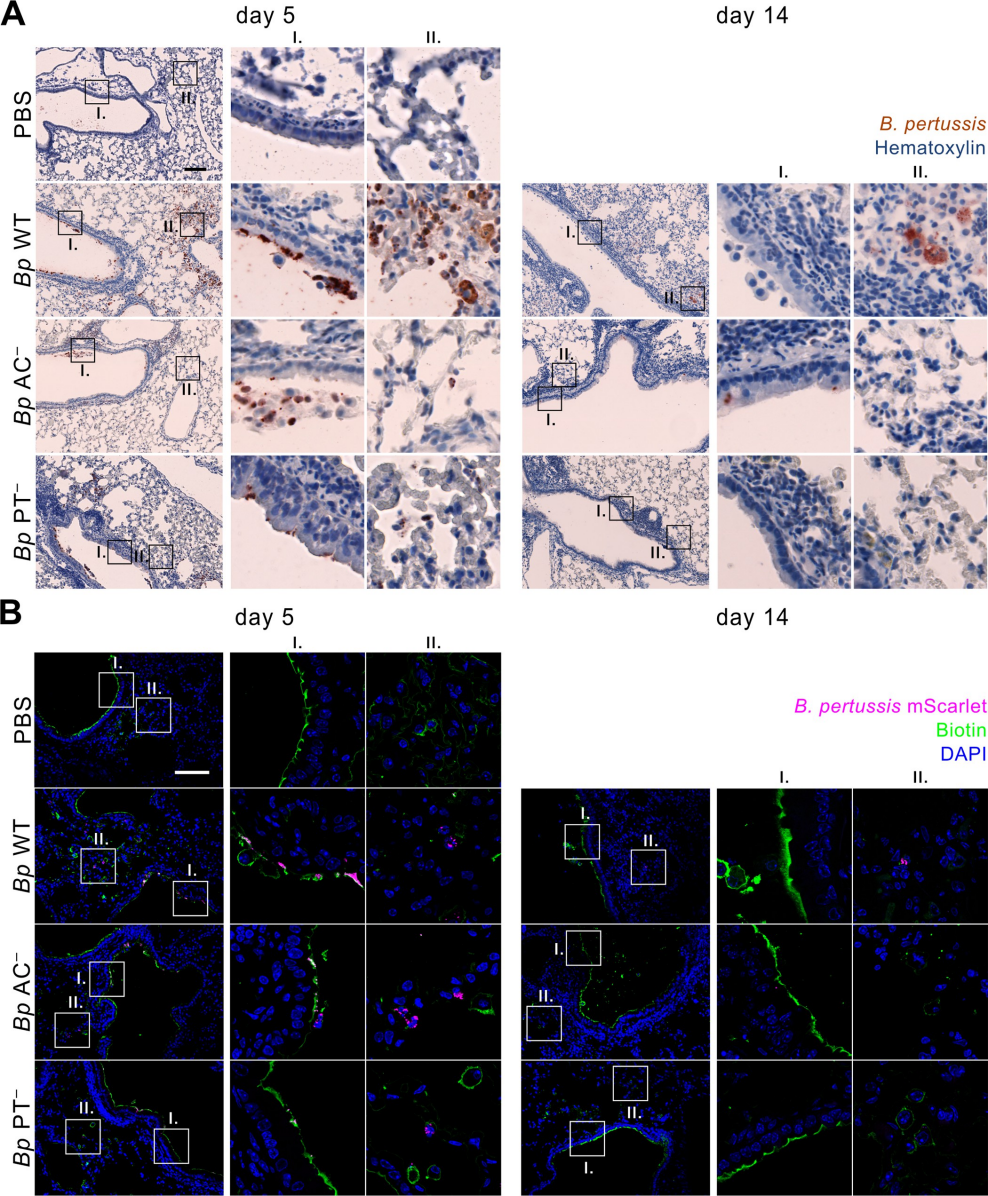
Enhanced *Bp* PT^−^ accumulation in mLNs is not due to enhanced penetration of *Bp* PT^−^ bacteria into lung parenchyma. **(A)** Immunohistochemistry of longitudinal lung sections from infected mice on day 5 (left panel) and day 14 (right panel) after intranasal administration of PBS (control) or 8 × 10^5^ CFU of the indicated *B*. *pertussis* mScarlet-producing strains. Lungs were fixed with 4% PFA, embedded in paraffin and examined upon immunohistochemical staining of 2 μm sections with polyclonal rabbit anti-*B*. *pertussis* serum. Details of bronchial epithelium (I.) and lung parenchyma (II.) are indicated. Data represent representative images of groups of three mice analyzed in two independent experiments. Scale bar 100 μm. **(B)** Epithelial lining of infected mouse lungs on day 5 (left panel) and day 14 (right panel). At indicated time points, the epithelial lining of the airways of mice was labeled *in vivo* by intranasal administration of 50 μl NHS biotin (1 mg/ml) for 5 minutes before the animals were euthanized. Lungs were fixed with 4% PFA, snap frozen and 10 μm longitudinal cryosections of the left lobes were labeled with Alexa Fluor 488 streptavidin conjugate. Images were acquired using a Leica TCS SPE confocal microscope. Nuclei were visualized by DAPI staining. Details of bronchial epithelium (I.) and lung parenchyma (II.) are indicated. Biotin, nuclei and bacteria are rendered in green, blue, and magenta colors, respectively. The images are representative of one experiment performed in groups of three mice. Scale bar 100 μm.

**Fig 3:**
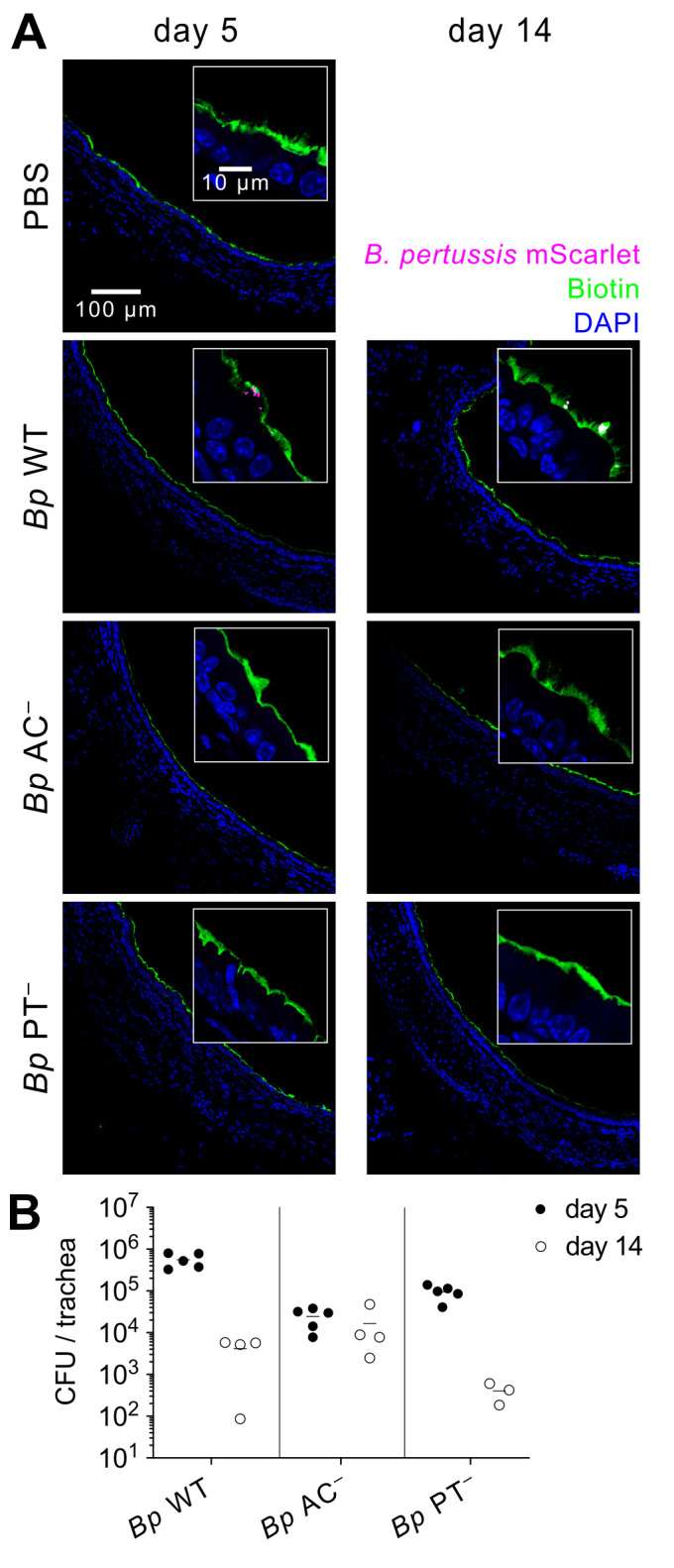
*Bp* PT^−^ does not cause enhanced disintegration of airway epithelial lining. **(A)** Tracheal epithelial lining of infected mouse lungs on day 5 (left panel) and day 14 (right panel). At indicated time points, the epithelial lining of the airways of mice was labeled *in vivo* by intranasal administration of 50 μl NHS biotin (1 mg/ml) for 5 minutes before the animals were euthanized. Respiratory tracts were fixed with 4% PFA, snap frozen and 10 μm transversal cryosections of tracheas were labeled with Alexa Fluor 488 streptavidin conjugate. Nuclei were visualized by DAPI staining. Images were acquired using a Leica TCS SPE confocal microscope. Biotin, nuclei and bacteria are rendered in green, blue, and magenta colors, respectively. The images are representative of one experiment performed in groups of three mice. Scale bar 100 μm. **(B)** Bacterial load in the trachea of infected mice on day 5 (full circles) and day 14 (open circles). Each symbol represents an individual animal, and the lines indicate the means. Data were pooled from two independent experiments.

**Fig 4:**
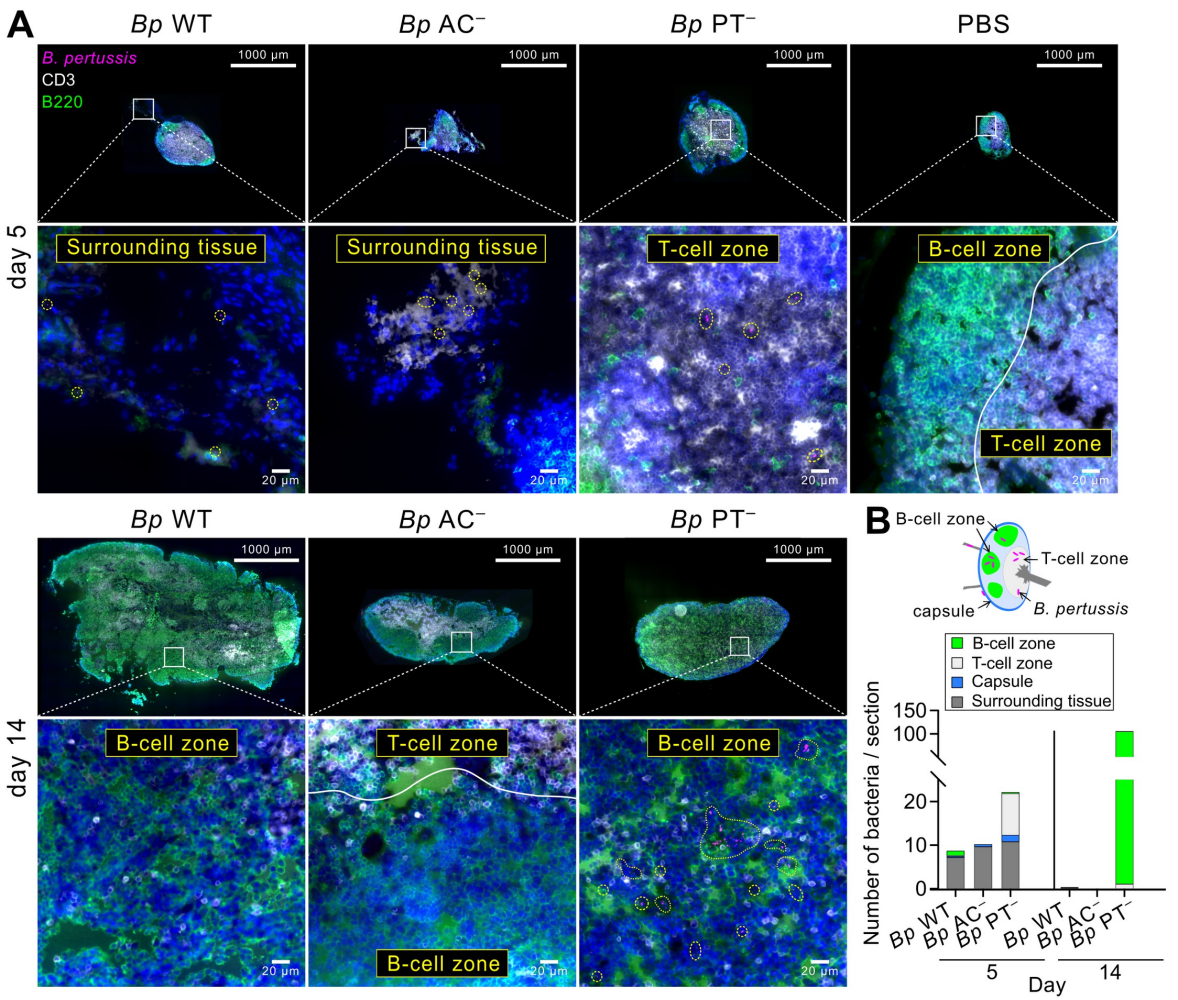
PT-producing *B*. *pertussis* does not enter the lymphoid tissue of mLNs, whereas *Bp* PT^−^ bacteria enter the T-cell zone and expand in the B-cell zone of mLNs. **(A)** Immunofluorescence microscopy of cryosections of mLNs of infected mice on days 5 (upper panel) and 14 (lower panel). mLNs were fixed with 4% PFA, snap frozen and 10 μm longitudinal cryosections were first labeled with rat anti-mouse CD45R (B220), followed by goat anti-rat Alexa Fluor 488 secondary antibody conjugate and finally by Alexa Fluor 647 rat anti-mouse CD3 antibody conjugate. Nuclei were labeled with DAPI. Stitched images were acquired at 40x magnification using an Olympus IX83 motorized automated microscope. Upper panels show representative images of entire mLN sections (scale bar 1000 μm). The white squares locate the zoomed-in areas shown in lower panels (see S6, S7, S8 and S9 Figs for high resolution images). Lower panels depict the details of the indicated areas (scale bar 20 μm). The mScarlet-expressing bacteria are encircled by yellow dotted lines. T cells, B cells, nuclei and bacteria are rendered in white, green, blue and magenta colors, respectively. In total 8 mLNs from three mice per infection group were examined and representative images are shown. **(B)** Location of bacteria on mLN sections. Individual bacteria were counted across entire mLN sections and location of bacteria in the indicated mLN zones (schematic drawing) was recorded. Mean bacterial counts of 6 mLN sections from 3 mice per infection group are shown.

**Fig 5:**
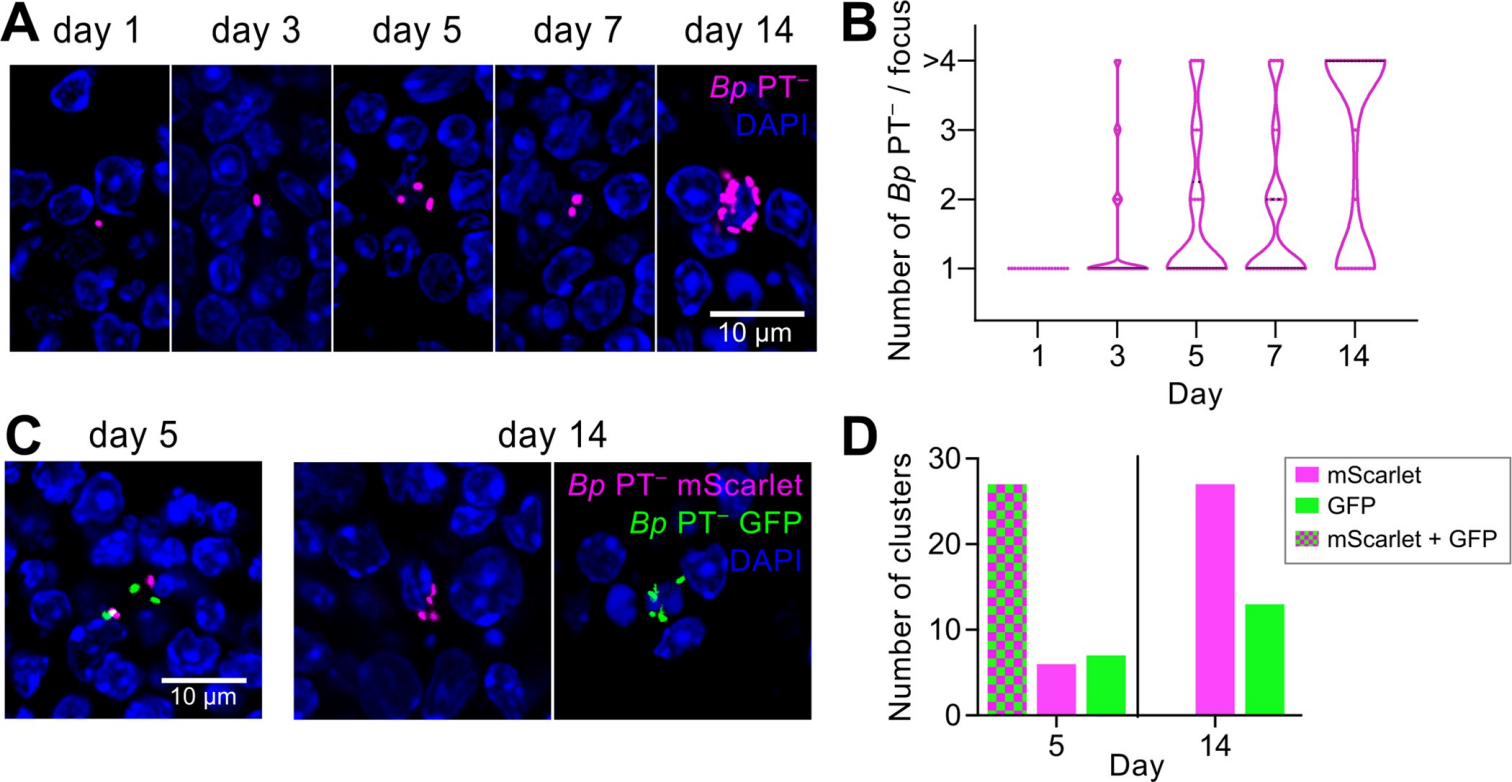
PT-deficient *B*. *pertussis* bacteria proliferate in mLNs. **(A)** Accumulation of mScarlet-producing *Bp* PT^−^ bacteria in the mLNs of infected mice. 30 μm cryosections of mLNs were examined by confocal microscopy. Scale bar 10 μm. **(B)** Quantification of bacterial clustering in the mLNs. Violin plots of the number of detected bacteria per site at the indicated time points. 30 randomly selected fluorescent foci containing at least one bacterium were examined per time point. Clusters containing more than 4 individual bacteria were categorized as > 4. Data were obtained from serial cryosections of at least three mLNs in one experiment. **(C)** Representative mLN cryosections (30 μm) from day 5 and 14 of mice infected with a 1:1 mixture of *Bp* PT^−^ strains producing mScarlet (magenta) and GFP (green) fluorescent proteins. Images were acquired at 63x magnification using a Leica TCS SPE confocal microscope. **(D)** Quantification of unicolor and multicolor clusters in mLNs of mice challenged with a 1:1 mixture of *Bp* PT^−^ strains producing mScarlet and GFP fluorescent proteins. 40 randomly selected bacterial clusters containing at least 2 individual bacterial cells were analyzed per time point. The predominance of mScarlet- over GFP-labeled unicolor clusters was not due to enhanced fitness but rather reflected easier microscopic detection of the brighter mScarlet-producing bacteria. Comparable numbers of magenta and green fluorescent colonies were recovered by plating of mLN homogenates on BG blood agar plates. Data were obtained from serial cryosections of at least six mLNs derived from groups of at least two mice from three independent experiments.

Following proliferation in the lungs for the first 3 days, the *Bp* PT^−^ mutant was rapidly cleared (*cf*. Fig 1A) and caused milder inflammatory damage of lung tissue (S3 Fig), thus being unlikely to be drained from the parenchyma into the mLNs in higher numbers than *Bp* WT bacteria. Indeed, higher numbers of *Bp* WT than *Bp* PT^−^ bacteria were visualized within lung parenchyma on day 5 of infection by immunohistochemistry and immunofluorescence microscopy (Fig 2A and 2B). Further, no difference to uninfected control airways was identified when observing penetration of intranasally applied NHS-biotin [72] into lung parenchyma (Fig 2B), or the lamina propria of tracheas (Fig 3A and 3B) in mice infected by the *Bp* WT or the *Bp* PT^−^ and *Bp* AC^−^ strains. We thus performed confocal fluorescence microscopy of mLN cryosections to assess whether the mScarlet-producing *B*. *pertussis* bacteria arrived into the lymphoid tissue of mLNs of infected animals (Figs 4 and S5–S10). Compared to the tiny mLNs of uninfected animals, mLN hypertrophy was observed on day 5 in animals infected by the *Bp* WT and *Bp* PT^−^ bacteria producing active ACT (Fig 4A, top), with striking mLN enlargement observed by day 14 (Fig 4A, bottom). The mScarlet-producing coccobacilli were observed on the cryosections as very bright fluorescent objects, likely corresponding to intact and viable bacteria present in the mLN tissue (Figs 4, S5 and S10). Bacterial cells were detected on day 5 sections of mLNs of all infected animals, but only the PT^−^ bacteria were abundantly present inside of the lymphoid tissue in all collected mLN samples (Figs 4A top, 4B, S7 and S10). In contrast, the PT-producing *Bp* WT and *Bp* AC^−^ bacteria were only detected outside of the mLNs on day 5, remaining attached to the mLN capsule, or being trapped within the surrounding non-lymphoid tissue, such as lymphatic vessels (Figs 4A, top, 4B, S6, S10). Hence, the PT-producing bacteria were drained from inflamed lungs by the lymphatics but were not delivered into the lymphoid tissue of the mLNs. Moreover, staining of the lymph node compartments for the CD3 (T-cell) and B220 (B-cell) markers revealed that the *Bp* PT^−^ bacteria localized mainly within the T-cell zone (paracortex) on day 5 (Figs 4A, top, 4B, S7 and S10), whereas on day 14, the *Bp* PT^−^ bacteria were mostly found in close contact with B-cells in the cortex (Figs 4A, bottom, 4B, S9 and S10). Furthermore, the *Bp* PT^−^ bacteria were not only found in deeper regions of the mLNs, but were also increasingly found in clusters by day 14 of infection, suggesting proliferation of the *Bp* PT^−^ bacteria within mLNs (Figs 4A, S9, S10, 5A, 5B and S11A). While single *Bp* PT^−^ cells were mostly observed in mLNs on days 1 and 3 after infection, groups of 2–3 bacterial cells, or even clusters of several *Bp* PT^−^ bacteria were found in mLNs on days 5 and 7. Abundant clusters of fluorescent *Bp* PT^−^ coccobacilli were then observed on all examined mLN sections from day 14 (Fig 5A and 5B), in line with the increase of CFU counts detected in mLN homogenates between days 7 and 14 (*cf*. Fig 1C). Single bacteria were still observed on day 14, but many cells with clusters of bacteria in the vicinity of their nuclei, as well as groups of extracellular bacteria scattered in the lymphoid tissue, were also observed (S11A Fig).

To test if the clusters of *Bp* PT^−^ bacteria arose by replication of individual founder bacteria, or through sequential phagocytic events, mice were infected with a 1:1 mixture of *Bp* PT^−^ bacteria expressing mScarlet (magenta) or GFP (green) fluorescent proteins (S1A, S5C, S5D and S11B Figs). As shown in Figs 5C, 5D and S11B, mixed groups of green and magenta fluorescent bacteria were observed 5 days after infection, likely resulting from sequential phagocytosis. However, no mixed color bacterial clusters were observed by day 14 anymore and unicolor fluorescent clusters were observed instead on a number of lymph node sections from 3 independent experiments, thus indicating clonal proliferation of the bacteria inside mLNs.

### PT-deficient *B*. *pertussis* is delivered from lungs into mLNs by migratory dendritic cells

**Fig 6:**
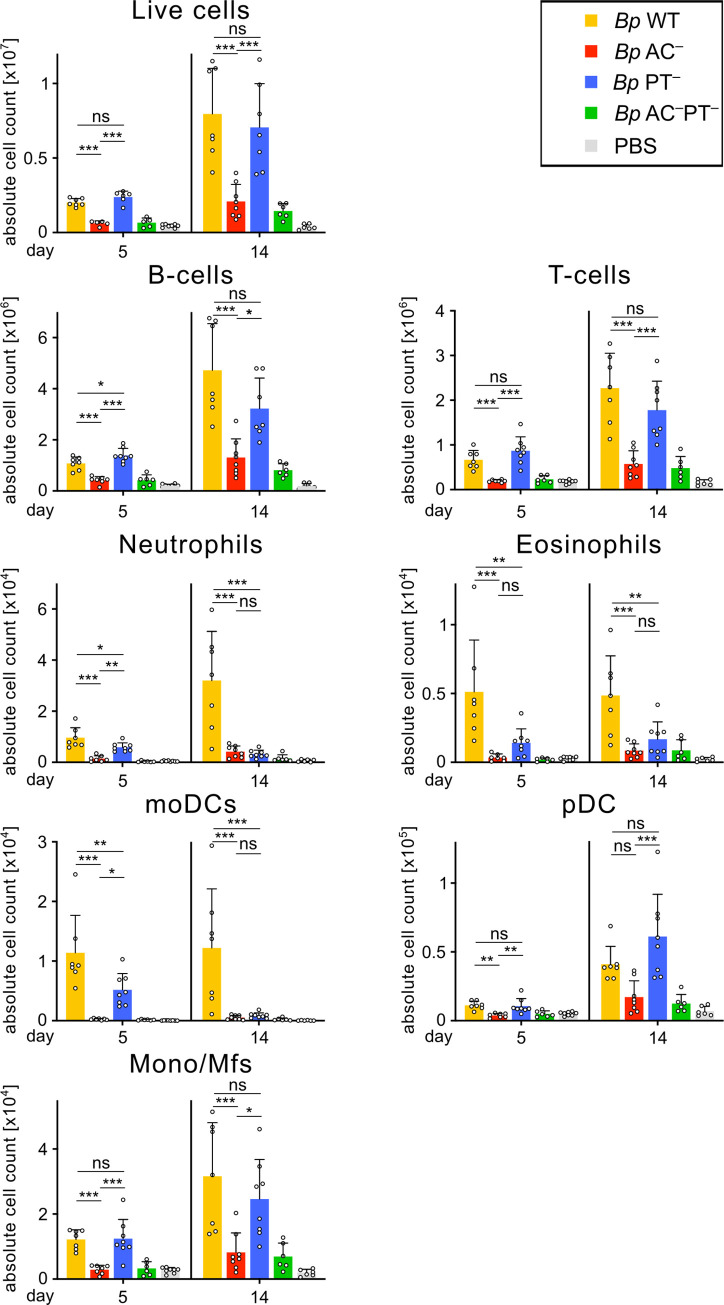
ACT activity drives immune cell accumulation in mLNs. Total counts of cells per mLN pool per infected mouse on day 5 and 14. Mice were intranasally infected with 8 × 10^5^ CFU of the indicated *B*. *pertussis* strains expressing mScarlet fluorescent protein. Collected mLNs were pooled, enzymatically disrupted and the cell suspensions were analyzed by flow cytometry using a panel of monoclonal antibodies and cell counting beads. Each symbol represents the value for an individual animal. Data represent mean values and standard deviations for groups of four mice per time point (or 2 mice in *Bp* AC^–^PT^−^ group) from two independent experiments. Statistical significance between groups was analyzed by one-way ANOVA followed by Tukey’s multiple comparisons test. * (p < 0.05), ** (p < 0.01), *** p (< 0.001). moDCs, monocyte-derived dendritic cells; pDCs, plasmacytoid dendritic cells; Mono/Mfs, monocytes/macrophages.

**Fig 7:**
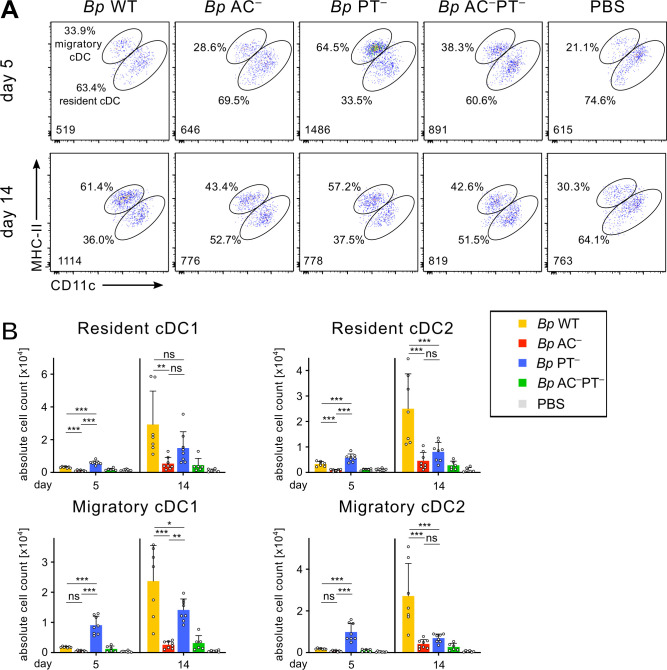
PT activity blocks early arrival of migratory cDCs from infected lungs to mLNs. Mice were intranasally infected with 8 × 10^5^ CFU of the indicated *B*. *pertussis* strains expressing mScarlet fluorescent protein. Collected mLNs were pooled, enzymatically disrupted and the cell suspensions were analyzed by flow cytometry using a panel of monoclonal antibodies and cell counting beads. **(A)** Representative dot plots of the migratory cDCs (MHC-II^high^CD11c^int^) and resident cDCs (MHC-II^int^CD11c^high^) detected in mLNs of infected mice on day 5 (upper panel) and 14 (lower panel). The indicated conventional dendritic cell (cDC) numbers (lower left corners) were gated-out from 100,000 viable singlets per sample as described in S12 Fig. The percentage (%) of cDC subpopulations are indicated. **(B)** Total counts of migratory and resident cDC1 (CD11b^–^) and cDC2 (CD11b^+^) cells in mLNs of infected mice on days 5 and 14. Each symbol represents the value for an individual animal. Data represent mean values and standard deviations for groups of four mice per time point (or 2 mice in *Bp* AC^–^PT^−^ group) from two independent experiments. Statistical significance between groups was analyzed by one-way ANOVA followed by Tukey’s multiple comparisons test. * (p < 0.05), ** (p < 0.01), *** p (< 0.001).

Since draining of wild-type *B*. *pertussis* from inflamed lung parenchyma by the lymphatics did not cause their accumulation in the lymphoid tissue of mLNs, we reasoned that *Bp* PT^−^ bacteria might be specifically delivered into mLNs by some migratory phagocytes known to traffic antigens for presentation in lymph nodes. Therefore, we analyzed the leukocyte composition of mLNs by multicolor flow cytometry. In line with the observed hypertrophy (*cf*. Figs 1B and 4A), the mLNs of mice infected by *Bp* WT and *Bp* PT^−^ bacteria contained significantly higher total numbers of cells than mLNs of *Bp* AC^–^-infected or uninfected animals (*cf*. Fig 6, top, see S12 for gating strategy). A generalized increase in numbers of all types of immune cells (*e*.*g*. B- and T-lymphocytes, neutrophils, eosinophils, Ly6c^high^ monocyte-derived DCs (moDCs) and CD11b^+^ monocytes and macrophages) was detected in the mLNs on days 5 and 14 of *Bp* WT and *Bp* PT^−^ infection, as compared to infection by the *Bp* AC^−^ bacteria producing active PT but inactive ACT (Fig 6). Intriguingly, lung infection by the *Bp* PT^−^ bacteria triggered a significant early increase of MHC-II^high^CD11c^int^ migratory conventional dendritic cells (cDC) in mLNs by day 5 of infection (Fig 7A and 7B). In turn, *Bp* WT bacteria, proliferating to much higher counts in the lungs, elicited a delayed increase of cDC numbers in mLNs by day 14 of infection. Furthermore, ~65% of all cDCs found in the mLNs on day 5 of *Bp* PT^−^ infection were migratory cDCs (Fig 7A), with roughly equal numbers of CD11b^–^ cDC1 and CD11b^+^ cDC2 migratory cells (Fig 7B). These results revealed that PT activity blocked early outmigration of cDCs from *B*. *pertussis*-infected lungs into mLNs and suggested that *Bp* PT^−^ bacteria may be delivered into mLNs by migratory cDCs capable of transporting various microorganisms [73–82].

**Fig 8:**
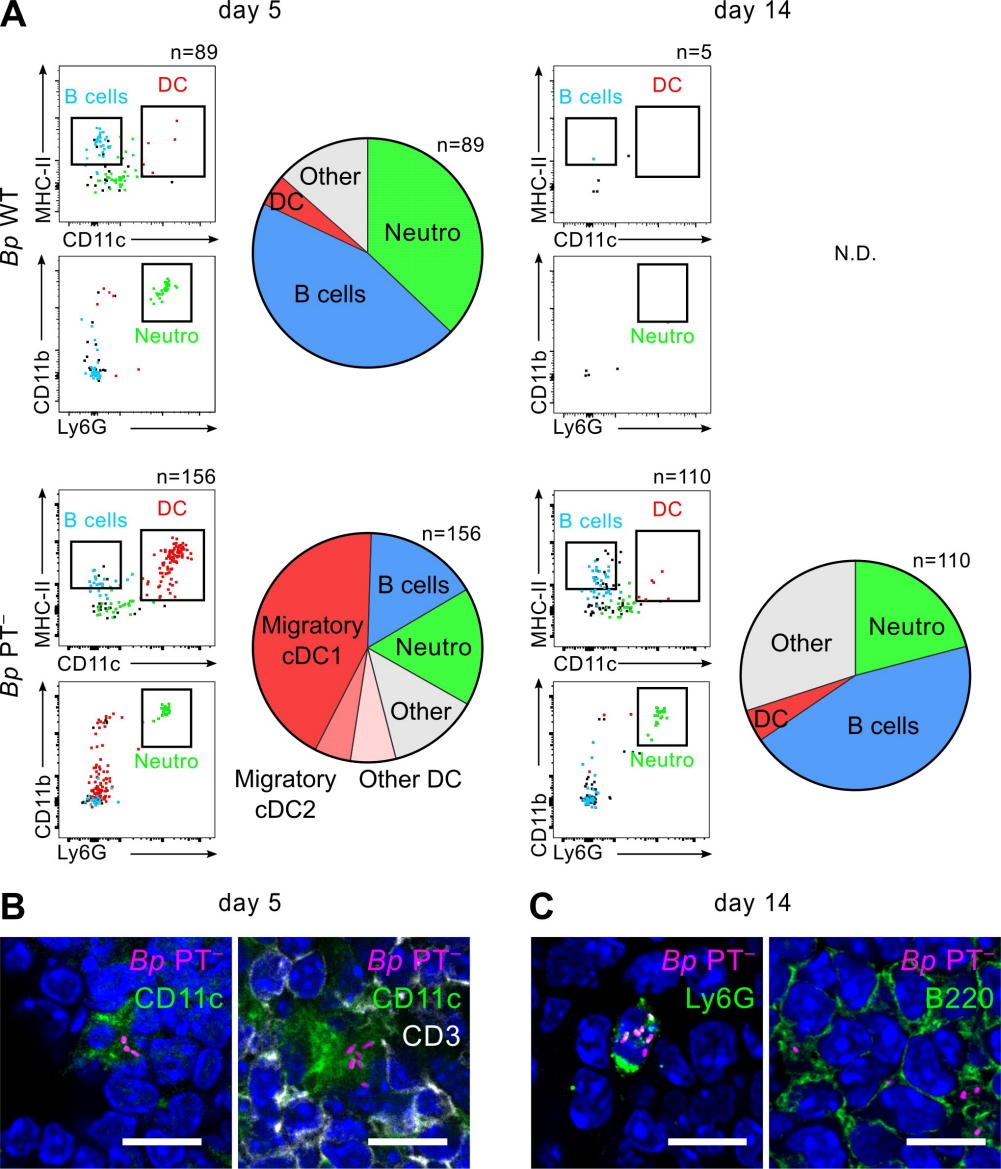
*Bp* PT^−^ bacteria are delivered into mLNs by migratory cDC1 cells. mLNs of mice infected with 50 μL of bacterial suspension containing 8 x 10^5^ CFU of the mScarlet^+^ *B*. *pertussis* strains were collected and pooled on days 5 and 14 using 4 mice per condition. **(A)** Cellular suspensions of mLNs were prepared by enzymatic disruption and cellular subpopulations were stained with a panel of fluorescently-labeled antibodies and analyzed by flow cytometry. The indicated numbers represent mScarlet^+^ cells detected per 2 x 10^6^ events on day 5 and 14, except for day 14 of the mLN sample of mice infected by *Bp* PT^–^, where the sample of analyzed cells was increased to 6 x 10^6^ events. mScarlet^+^ cells associated with *Bp* bacteria were detected using 585/15 nm emission filter and the gate was set using cellular suspension of mLNs from mice infected with the non-fluorescent control bacteria (S14 Fig). *Bp*-associated cells were visualized in simple dot plots from down-sampled live mScarlet^+^ events (left panels): MHC-II^+^ CD11c^+^ dendritic cells (DC, red dots), CD19^+^CD3^–^ B cells (blue dots, S14 Fig) and CD11b^+^Ly6G^+^ neutrophils (green dots). Pie charts on the right-hand panels show the distribution of subpopulations of mScarlet^+^ cells determined by in depth phenotyping (see S12 Fig for gating strategy). Data from one representative experiment out of 3 (*Bp* WT) or 4 (*Bp* PT^–^) performed are shown. **(B)** and **(C)** In parallel to cytometric analysis, cryosections of mLNs from *Bp* PT^–^-infected mice were prepared on days 5 and 14 and mScarlet^+^ cells were visualized by immunofluorescence microscopy. **(B)** CD11c^+^ dendritic cells were detected on day 5 with biotin-conjugated anti-CD11c followed by AF488-conjugated streptavidin (green). T cells were detected with AF647-labeled anti-CD3 (white). **(C)** on day 14, neutrophils were detected with biotin-conjugated rat Ly6G antibody followed by AF488-conjugated streptavidin (green) and B cells were detected with rat anti-B220 antibody followed by goat anti-rat AF488-labeled secondary antibody (green). Images were acquired at 63x magnification using Leica TCS SPE confocal microscope. Scale bar 10 μm.

To better identify the cells harboring the mScarlet-expressing bacteria, flow cytometry of mLN suspensions was used. This was technically challenging, as only ~15% of the low numbers of viable bacteria detected in the mLNs (*cf*. Fig 1C) were associated with pelleted cells upon low speed (300 x *g*) centrifugation of mLN homogenates and the rest of the bacteria appeared to be extracellular (S13 Fig). Hence, a very low proportion of mLN cells was expected to contain fluorescent bacteria. Therefore, autofluorescence and unspecific antibody staining had to be rigorously controlled by parallel processing of mLN cells from animals infected by corresponding non-fluorescent *B*. *pertussis* strains. Such comparative cytometric analysis revealed that mScarlet^+^ events were about ten-times more frequent than unspecific events (S14 Fig). Upon collection of 10^6^−10^7^ events, dozens to hundreds of mScarlet-positive cells were reliably detected, thus representing ~0.002–0.01% of live cells in mLN suspensions (S14 Fig). As depicted on Figs 8A and S14, the mScarlet^+^ cells detected in mLNs from *Bp* WT-infected mice on day 5 phenotyped as CD19^+^ MHC-II^+^ B cells (~45%) and Ly6G^+^ CD11b^high^ neutrophils (~37%), with few infected DCs (~4%) detected. In contrast, DCs reproducibly made up the largest share of *Bp* PT^–^-infected cells. The mScarlet^+^ *Bp* PT^−^ bacteria were mostly associated with migratory CD11b^–^ cDC1 cells (43%), migratory CD11b^+^ cDC2 cells (5%), or other dendritic cell (6%) populations. Fluorescence microscopy of mLN cryosections revealed that such *Bp* PT^–^-harboring CD11c^+^ cells of dendritic shape were often in contact with CD3^+^ T-cells (Figs 8B, see S15 for more images). These results indicate that by day 5 of infection, the *Bp* PT^−^ bacteria were transported from the infected lungs into mLNs mostly by migratory cDC1 cells. Later, by day 14 of infection (Figs 8A and S14), ~21% of cells containing mScarlet^+^ bacteria were polymorphonuclear Ly6G^+^ neutrophils, often harboring multiple intracellular *Bp* PT^−^ bacteria (Figs 8C, see S16 for more images), while a larger fraction (~45%) of mScarlet^+^ *Bp* PT^−^ bacteria were associated with CD19^+^MHC-II^+^ B cells (Fig 8A) located in the B (B220^+^) cell zone on mLN cryosections by day 14 of infection (*cf*. Figs 4A, 8C, S9 and S10).

### PT-deficient *B*. *pertussis* infection triggers a very early serum antibody response

**Fig 9:**
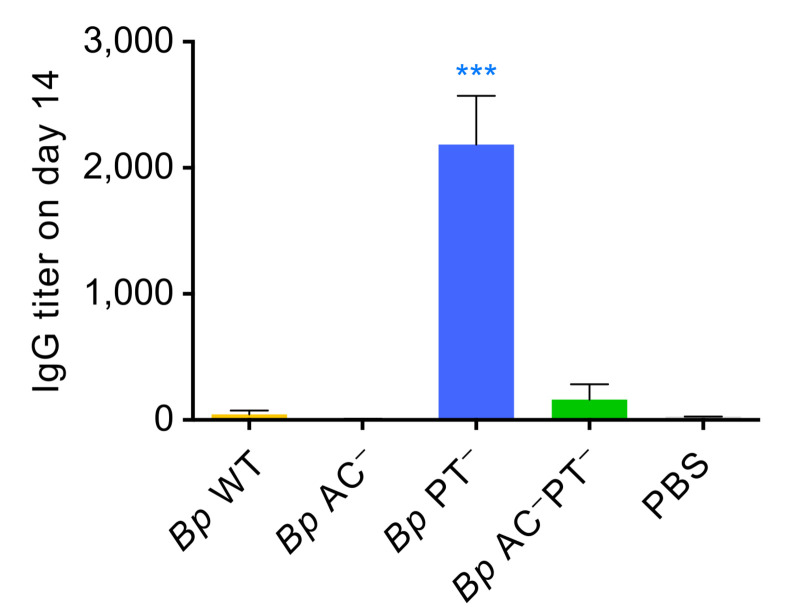
PT action prevents early adaptive immune response to *B*. *pertussis* infection. Sera of mice were collected 14 days after infection with 8 x 10^5^ CFU of the indicated *B*. *pertussis* strains (mScarlet^+^). Total anti-*B*. *pertussis* IgG antibody titters were determined by whole bacterial cell ELISA using plates coated with heat-killed *B*. *pertussis* (Tohama I). Mean antibody titers were determined as the inflection points of titration curves +/- SD. Pools of sera from 2 mice from two independent experiments (total n = 4 mice/group) were analyzed in technical triplicates. Groups were compared to *Bp* WT using one-way ANOVA with Dunnett’s multiple comparisons test. * p < 0.05, ** p < 0.01, *** p < 0.001, **** p < 0.0001.

The above results indicated that PT action prevents the delivery of *B*. *pertussis* antigens into mLNs by migratory DCs and this might delay induction of the adaptive immune response to infection. We thus compared the levels of *Bp*-specific IgG antibodies in sera collected from the infected mice as early as 14 days after bacterial inoculation (Fig 9). Intriguingly, the sera of *Bp* PT^–^-challenged mice contained a substantial level of *B*. *pertussis*-specific IgG antibodies, exhibiting a titer of ~2,000 already 14 days after infection. In contrast, no anti-*B*. *pertussis* IgG was detectable in sera collected 14 days after infection from mice inoculated by the PT-producing wild-type or *Bp* AC^−^ mutant bacteria that proliferated in mouse lungs to substantially higher levels and persisted longer than the *Bp* PT^−^ bacteria. In line with these antibody responses, already on day 5 of infection the splenocytes of mice infected with *Bp* PT^−^ responded to antigenic restimulation by higher production of inflammatory cytokines (*e*.*g*. IFNγ, TNF-α, IL-6 and IL-12p70) than splenocytes of mice infected by the PT producing wild-type bacteria (S17 Fig). Intriguingly, some *B*. *pertussis*-specific IgG were also detectable in sera of mice infected by the *Bp* AC^–^PT^−^ double mutant despite its rapid clearance from the lungs.

## Discussion

We report that PT activity is critical for prevention of migratory dendritic cell-mediated delivery of *B*. *pertussis* bacteria from the infected lung parenchyma into the lymphoid tissue of lung-draining mediastinal lymph nodes (mLNs). The PT-producing bacteria largely failed to enter the mLNs and remained trapped within the associated tissue outside of mLNs, or localized to the capsule or the subcapsular sinus, as expected for bacteria being simply shuttled from the infected lung parenchyma by the lymph. In contrast, mLN cryosection microscopy revealed that in the absence of PT activity the cDC-associated *Bp* PT^−^ bacteria were delivered deep inside the T-cell zone (paracortex) of the LN, which is a zone accessed by migratory DCs delivering antigens for presentation to T cells. This indicates that the PT^−^ bacteria were readily transported by migratory cDCs into deeper regions of mLNs. While being almost cleared from the infected lungs by day 14, the ACT-producing PT^−^ bacteria proliferated in mLNs by that time to higher counts than found in the lungs and relocated to the B-cell zone (cortex) of mLNs, where they often formed clusters. This suggests that the capacity of ACT to ablate the oxidative burst of phagocytes, block killing of phagocytosed non-opsonized bacteria and trigger apoptosis by cAMP-mediated signaling [34,35,41] enabled the release of live bacteria into the lymphoid tissue of mLNs from the apoptosing cDCs [83]. Indeed, when mice were infected with a mixture of green and magenta fluorescent *Bp* PT^−^ bacteria, dual-labeled groups of phagocytosed bacteria were still observed in the LNs on day 5. However, on day 14 only unicolor bacterial foci were observed, revealing that each bacterial cluster originated by proliferation of a single founder bacterium inside the mLNs. Nevertheless, the PT^−^ bacteria did not disseminate beyond mLNs to other organs within 14 days of infection and colonized the mLNs only transiently. By day 21 (*cf*. S4 Fig), the CFU counts in mLNs started to decline and viable PT^−^ bacteria were nearly absent from the mLNs 5 weeks after infection (~10^0^−10^1^ CFU), likely due to onset of antibody-dependent opsonophagocytic killing.

The inhibition of cDC-mediated delivery of PT-producing bacteria into the mLNs would be in line with PT-mediated perturbation of G_i/o_-associated chemokine receptor signaling (*e*.*g*. CCR7) and with the *in vitro* observed PT-dependent inhibition of dendritic cell migration towards the lymphoid chemokine CCL21 [69]. Inhibition of delivery of *B*. *pertussis* bacteria into the LNs by PT would also suggest why the bacterium generally does not cause disseminated systemic infections in immunocompetent humans [84,85] despite being able to proliferate in the infected upper airways of human infants to as high levels as up to ~10^8^ *B*. *pertussis* CFU/ml of undiluted nasal aspirates [86]. Moreover, a striking influx of neutrophils into mLNs of *Bp* WT-infected animals was observed on day 14, suggesting a role of neutrophils in clearance of *B*. *pertussis* from the lymphatic system (*cf*. Figs 6 and 8).

In contrast, the *B*. *bronchiseptica* species, which shares a common ancestor with *B*. *pertussis* and produces ACT but not PT [87], was previously found to infect the NALT and mLNs of experimentally infected mice [88] and was reported to cause disseminated infections in immunocompromised humans [89,90]. Indeed, *B*. *bronchiseptica* triggers an early migration of DCs from the infected airways into the mLNs [88]. Moreover, *B*. *holmesii* that does not produce any of the classical virulence factors (PT, ACT, Type III secretion systems, pertactin or fimbriae) is still able to cause a disseminated infection and pertussis-like illness with a much higher capacity of invasiveness into other tissues than *B*. *pertussis* [91].

The delivery of PT-deficient bacteria into mLNs indicates that one of the biological roles of PT consists in restriction of *B*. *pertussis* dissemination beyond the mucosa by phagocytic cells, so as to limit the early immune response to infection. In this respect, *B*. *pertussis* differs importantly from many other pathogens that exploit cDCs as a Trojan horse for delivery of live microorganisms into mLNs to cause systemic infections that are part of their natural life cycle [75,77–80]. In the case o*f B*. *pertussis*, such hijacking of cDCs by the *Bp* PT^−^ bacteria devoid of PT activity appears as counterproductive. Delivery of PT-deficient *Bp* PT^−^ bacteria into mLNs ramped up the immune response to *Bp* PT^−^ infection. Indeed, it has previously been shown for *M*. *tuberculosis* that the mere presence of bacteria in close proximity to lymphocytes within the LNs can boost antigen presentation and the overall immune response to infection [92]. It will be of interest to examine if also the PT^−^ *B*. *pertussis* BPZE1 bacteria are trafficked into mucosa-draining LNs and whether this enhances the immunogenicity of the intranasally applied live attenuated BPZE1 pertussis vaccine, which has already passed phase IIb clinical trials in adults and advances into trials in school age children (https://www.iliadbio.com/clinical.html).

We hypothesized previously that the cAMP signaling activity of ACT might drive outmigration of immature intraepithelial DCs into draining LNs in the course of *B*. *pertussis* infection, as ACT action was found to hamper maturation of DCs while increasing their LPS-stimulated chemotactic migration *in vitro* [46]. Indeed, on day 5 of lung infection with the ACT-producing *Bp* PT^−^ bacteria we observed a significant increase in the absolute numbers, as well as in the relative proportion, of migratory conventional DCs (both CD11b^-^ cDC1 and CD11b^+^ cDC2) in the lung-draining mLNs (*cf*. Fig 7A and 7B). These CD11b^–^ cDC1 likely corresponded to the intraepithelial DC population [93,94] and the most prominent cell type associated with the *Bp* PT^−^ bacteria on day 5 in the mLNs was phenotyped as MHC-II^high^CD11c^int^ migratory cDC1 (CD11b^–^) cells. However, such cDC number increase in mLNs was not observed upon lung infection by the PT-producing wild-type bacteria that proliferated in the lungs to importantly higher levels. This suggests that PT action inhibited the LOS/TLR4-elicted and ACT-potentiated chemotactic cDC outmigration from the site of infection. Delaying the delivery of bacterial antigens for presentation to B and T cells in the lymph nodes would then likely delay the induction of an early adaptive immune response to infection. Indeed, others have observed in mouse models that anti-*B*. *pertussis* antibody responses appear in only ~3–4 weeks, or even later upon infection by PT-producing wild-type *B*. *pertussis*, later when the bacteria are already being cleared from the infected lungs through antibody-dependent phagocytosis by neutrophils [52,95–97]. We found that upon infection by the *Bp* PT^−^ mutant a robust serum IgG antibody response to *B*. *pertussis* infection was detected as early, as on day 14 after infection. It is, hence, plausible to assume that the PT-provoked delay in delivery of bacterial antigen into LNs serves to delay the induction of antibody response to infection, in line with a previous observation that PT action suppressed serum antibody responses to immunodominant *B*. *pertussis* antigens [53]. This would enable *B*. *pertussis* to proliferate to the observed high levels on the mucosa of the upper airway during the catarrhal phase of infection and would support its efficient aerosol-mediated transmission to new hosts.

## Materials and methods

### Ethics statement

All animal experiments were approved by the Animal Welfare Committee of the Institute of Molecular Genetics of the CAS, v.v.i. in accordance with the Guidelines for the Care and Use of Laboratory Animals, the Act of Czech National Assembly, the Collection of Laws no. 246/1992. Permissions no. 47/2016 and 10/2020 were issued by the Animal Welfare Committee of the Institute of Molecular Genetics of the Czech Academy of Sciences in Prague.

### Antibodies and reagents

The antibodies used in this study are listed in S1 Table. Rabbit anti-*B*. *pertussis* serum was kindly provided by Branislav Vecerek (Institute of Microbiology, Prague, Czech Republic). Anti-ACT (clone 9D4) monoclonal antibody was provided by Erik Hewlett (University of Virginia, USA). The monoclonal antibody against the S1 subunit of pertussis toxin (clone 63.1G9) was purchased from Santa Cruz Biotechnology (USA). EZ-Link Sulfo-NHS-Biotin was from Thermo Fisher Scientific (USA).

### Plasmid construction

The mScarlet [70] and superfolder green fluorescent (sfGFP) [98] proteins were produced in *B*. *pertussis* using pBBR1-derived [99] vectors carrying the *mScarlet* or *sfGFP* genes under the control of BvgAS-regulated filamentous hemagglutinin (*fhaB* gene) promoter (P*fhaB*) [100] (S1A and S1B Fig), as follows. The pBBR1-mScarlet plasmid was obtained by insertion of a P*fhaB* promoter fragment amplified by PCR from *B*. *pertussis* genomic DNA using the primers 5’-TTTGAGCTCGGCCGGGGAGCGGGTTG-3’ and 5’-TTTATGCATATGTATATCTCCTTCTTAAATCTAGAGGATCCGTTATCGGCCTGCTCG-3’, digested by SacI and NsiI enzymes and ligated into the SacI/BamHI-cleaved pBRR1 vector together with a NsiI/BamHI-digested PCR fragment carrying the synthetic mScarlet gene amplified from the pmScarlet_C1 vector (Addgene #85042) using the primers 5’-TTTATGCATGTGAGCAAGGGCGAGGC-3’ and 5’-TTTGGATCCTTATCCGGACTTGTACAGCTC-3’. The pBBR1-sfGFP vector was prepared by insertion of the NdeI/PstI-digested PCR fragment carrrying the *sfGFP* gene amplified from the sfGFP-C1 vector (Addgene #54579) using primers (5’-TTTCATATGGCTAGCACTAGTGTG-3’ and 5’-TTTCTGCAGTCACTTGTACAGCTCGTCCATG-3’) into the NdeI/PstI-digested pBBR1-mScarlet vector. The plasmids were introduced into *B*. *pertussis* cells by conjugation, using *E*. *coli* SM10 λpir as plasmid donor strain and the *B*. *pertussis* exconjugants were selected on Bordet-Gengou (BG) blood agar plates supplemented with 10 μg/ml chloramphenicol and 100 μg/ml cephalexin.

### Bacterial strains and growth conditions

Construction of *B*. *pertussis* Tohama I (Institute Pasteur collection #CIP 81.32) mutants (*Bp* AC^–^, *Bp* PT^−^ and *Bp* AC^–^PT^–^), producing individually or in combination the enzymatically inactive adenylate cyclase toxoid (AC^–^) and the enzymatically inactive pertussis toxoid (PT^–^) (Table 1), was described previously [44]. *B*. *pertussis* strains were grown at 37°C on BG agar (Difco, USA) supplemented with 15% defibrinated sheep blood (LabMediaServis, Jaromer, Czech Republic) in a humidified 5% CO_2_ atmosphere for 3–7 days. Liquid cultures were grown at 37°C in modified Stainer-Scholte medium supplemented with 5 g/l Bacto Casamino Acids (Difco, USA) and 1 g/l heptakis (2,6-di-O-dimethyl) β-cyclodextrin (Sigma, USA). 10 μg/ml of chloramphenicol was added into media used for growth of the fluorescent *B*. *pertussis* strains carrying the pBBR1-mScarlet and pBBR1-sfGFP plasmids.

### Animal experiments

Animals were anesthetized prior challenge by intraperitoneal (i.p.) injection of ketamine (80 mg/kg) plus xylazine (8 mg/kg) and groups of three to six female BALB/cByJ mice (Charles River, Ecully, France) at 4–5 weeks of age were challenged intranasally with 50 μl of suspensions of *B*. *pertussis* cells containing 8 × 10^5^ colony-forming units (CFU). For the mixed infection experiment, a 1:1 mixture of *B*. *pertussis* cells (8 × 10^5^ CFU in total) expressing mScarlet and GFP proteins was used as described [101]. At the indicated time points (2 h, 3, 5, 7, and 14 days after challenge), mice were sacrificed by cervical dislocation, and the lungs, trachea, spleen and thymus were aseptically removed and homogenized in PBS using a tissue grinder (Heidolph RZR 2020, Merck, Germany). The removed mediastinal lymph nodes (mLN), cervical lymph nodes (cLN), and inguinal lymph nodes (iLN) were gently homogenized in cold PBS by passage through a Corning 70 μm cell strainer (Corning, USA). The numbers of viable bacteria in mouse organs were determined as colony-forming units (CFU) upon plating of tissue homogenates on BG agar plates. The percentage of non-fluorescent bacteria in the organs was determined by counting of fluorescent and non-fluorescent colonies using a G:Box Chemi XRQ gel doc system (Syngene, UK).

### Flow cytometry

Lymph nodes were mechanically disrupted by needles and homogenized in high glucose Dulbecco’s Modified Eagle’s medium (DMEM, Sigma) by enzymatic digestion with 2 mg/ml of collagenase D and 40 μg/ml of DNase I (Roche) at 37°C for 30 min. EDTA (14 mM) was added and cell suspensions were filtered through a 50 μm CellTrics strainer (Sysmex Partec, Germany). Prior to flow cytometric analysis, the cells were blocked with 10% BALB/c mouse serum and anti-mouse CD16/CD32 monoclonal antibody (12.5 μg/ml; eBioscience) in wash buffer for 30 min on ice and stained with fluorochrome-labeled monoclonal antibodies (S1 Table) and fixable viability dye eFluor 780 (eBioscience) for 30 min on ice in the dark. Where appropriate, CountBright Absolute Counting Beads (Invitrogen, USA) were added for determination of absolute cell counts. Cytometric data were acquired on a BD LSRII flow cytometer (BD Biosciences, USA) and analyzed using FlowJo v10 software (Tree Star, USA). The compensation matrix was calculated using single stain controls. To detect fluorescent bacteria associated with cells, live cells from tissues of mice infected with fluorescent or nonfluorescent bacteria were downsampled, using a DownSample FlowJo plugin to obtain identical numbers of cells, and then gated based on mScarlet fluorescence (585/15 nm emission filter).

### Histology and immunohistochemistry

Mice were sacrificed at indicated time points, the tracheas were cannulated with a 26-gauge needle and the lungs were inflated with 1 ml of 4% (w/v) PBS-buffered formaldehyde (PFA) solution. The entire respiratory tract and the mLNs were excised, placed in 4% PFA for 24 hours, transferred into 70% (vol/vol) denatured ethanol and dehydrated with ethanol series using a Leica ASP 6025 tissue processor (Leica Biosystems, Germany). The samples were embedded in paraffin using a Leica EG1150 C embedding system (Leica Biosystems, Germany) and cut to 2 μm-thick sections using a Leica RM2255 rotary microtome (Leica Biosystems, Germany). Hematoxylin and eosin staining (HE) was performed using an automated Leica ST5020 multistainer (Leica Biosystems, Germany). For immunohistochemical (IHC) staining of *B*. *pertussis* cells, tissue sections were rehydrated and antigen was retrieved at 95°C for 15 min at pH 6.0 with antigen retrieval solution (Zytomed Systems, Germany). Endogenous peroxidase was blocked with 3% (v/v) hydrogen peroxide in methanol. Sections were incubated with a rabbit anti-*B*. *pertussis* serum diluted 1:1,000 overnight at 4°C, followed by a 30-min incubation with the secondary anti-rabbit antibody conjugated to horseradish peroxidase (Zytomed Systems, Germany) at 25°C. Signal was developed in 3-amino-9-ethylcarbazole (AEC) solution (Zytomed Systems, Germany) for 10 min at 25°C. Sections were counterstained with hematoxylin (Sigma, USA), mounted with Aquatex mounting medium (Merck-Millipore, Germany), and scanned with an AxioScan.Z1 automatic slide scanner at 40x magnification (Carl Zeiss, Germany). Images were processed using ZEN software (Carl Zeiss, Germany).

### Immunofluorescence microscopy of frozen tissue sections

Immunofluorescence analysis of lungs, trachea, and lymph nodes was performed at indicated time intervals upon intranasal challenge of mice with PBS (control) or *B*. *pertussis*. For *in vivo* epithelial barrier permeability assessment, 50 μl of NHS-biotin in PBS (1 mg/ml) was intranasally applied to anesthetized mice for 5 min. Mice were euthanized by cervical dislocation, their lungs were inflated with 1 ml of 4% PFA, excised, and placed into 4% PFA for 24 h at 4°C. The PFA-fixed tissues were transferred into PBS-buffered 30% sucrose for 24 h at 4°C, embedded in a Tissue-Tek OCT cryo-embedding medium (Sakura Finetek, USA) and frozen in liquid nitrogen-cooled isopentane. The cryomolds were cut into 10 or 30 μm thick sections using a Leica CM1950 cryotome (Leica Biosystems, Germany) and were mounted on SuperFrost Plus adhesion slides (Thermo Fisher Scientific, USA) and stored at -20°C. Before staining, the slides were thawed at 25°C for 1 h and washed with PBS-T (PBS with 0.05% Tween 20). After blocking (5% [w/v] BSA in PBS or 5% [v/v] goat serum in PBS for goat secondary antibodies) and permeabilization (PBS supplemented with 0.3% [v/v] Triton X-100), the cryosections were stained overnight at 4°C with unconjugated, fluorochrome- or biotin-conjugated primary antibodies (antibody details in S1 Table) diluted in PBS containing 0.2% (w/v) BSA, followed by incubation at 20°C with the corresponding fluorochrome-conjugated secondary antibodies and Alexa Fluor-conjugated streptavidin (Thermo Fisher Scientific, USA) for 1 h and 30 min, respectively. *In vivo* biotinylation of tracheal epithelial cells was visualized directly by incubating the cryosections with Alexa Fluor 488-conjugated streptavidin (Thermo Fisher Scientific, USA). *B*. *pertussis* cells were labeled with rabbit anti-*B*. *pertussis* serum (diluted 1:5,000) followed by goat anti-rabbit Alexa Fluor 647-conjugated secondary antibody. The cell nuclei were stained with DAPI. Specificity of staining was verified by using the appropriate immunoglobulin isotype controls. Specimens were mounted in Vectashield Antifade Mounting Medium (Vector Laboratories, USA) and imaged using a Leica TCS SPE confocal microscope (Leica Biosystems, Germany). Stitched images of entire LN tissue sections were acquired at 40x magnification using an IX83 motorized and automated fluorescence microscope (Olympus, Japan) with cellSens software (Olympus, Japan). The images were processed using Fiji [102]. Where appropriate, background inhomogeneity (shading) was corrected using the plugin BaSiC [103].

### Immunodetection of secreted proteins

Proteins secreted into *B*. *pertussis* culture supernatants (13,000 x *g*, 5 min) were probed in immunoblots with appropriate monoclonal antibodies recognizing the ACT and PT proteins, decorated with horseradish peroxidase-conjugated goat anti-mouse secondary antibody (GE Healthcare, USA) and detected using the SuperSignal West Femto substrate (Thermo Fisher Scientific, USA) in a G:Box Chemi XRQ Gel Doc System (Syngene, UK).

### IgG level determination

Serum samples were prepared by centrifugation of whole blood at 5,000 × *g* at 4°C for 15 min after retro-orbital bleeding of anesthetized mice. Total IgG levels against *B*. *pertussis* antigens were determined using a whole bacterial cell ELISA as previously described [11] and the antibody titers were calculated as inflection points of titration curves.

### Statistical analysis

Statistical significance between groups was determined by one-way or two-way analysis of variance (ANOVA) followed by Dunnett‘s or Tukey’s post hoc tests. Differences were considered statistically significant at * p < 0.05, ** p < 0.01, *** p < 0.001, **** p < 0.0001.

## Supporting information

S1 Data Excel spreadsheet containing, in separate sheets, the underlying numerical data for Figure panels 1A, 1C, 1D, 3B, 4B, 5B, 5D, 6, 7B, 8A, 9, S2, S4, S13A, S13B, S17. (XLSX)

S1 Table List of antibodies used for flow cytometry and immunofluorescence staining of cryosections. IF (immunofluorescence), A700 (Alexa Fluor), PerCP (Peridinin-chlorophyll-protein Complex), PE-Cy7 (Phycoerythrin-Cyanine7), APC (Allophycocyanin), Af488 (Alexa Fluor), eF450 (eFluor), APC-eF780 (Allophycocyanin-eFluor), FITC (Fluorescein Isothiocyanate).
(TIF)

S1 Fig *In vitro* characterization of *B*. *pertussis* strains producing the mScarlet fluorescent protein. **(A)** Scheme of the pBBR1-derived plasmids for mScarlet and sfGFP production under the Bvg A/S-regulated P*fha* promoter control. Cm^R^ chloramphenicol resistance gene. **(B)** Stability of mScarlet fluorescent protein production in liquid culture. Bacteria were grown at 37°C in 3 ml of modified SS medium supplemented with 50 or 100 mM MgSO_4_, and in the presence or absence of 10 μg/ml chloramphenicol for 24 hours. Afterwards the culture was diluted to OD_600_ = 0.2 in the same medium and the bacteria were grown for another 24 hours. Fluorescence intensity at 605 nm was determined every 24 h in cultures diluted to OD = 0.2. **(C)** Growth curves of the used *B*. *pertussis* strains. Bacteria were grown in liquid cultures in 10 ml of modified SS medium without antibiotics at 37°C. OD_600nm_ was measured in appropriately diluted cultures at indicated time points. Means +/- SD from one experiment performed in duplicate are shown. Data are representative of three independent experiments. **(D)** Toxin or toxoid production by the used strains. Bacteria were grown without antibiotics in SS medium containing 2 mM CaCl_2_ to facilitate ACT secretion [106]. Culture supernatants were collected at OD = 1 by centrifugation and probed for ACT and PT antigen by Western blotting. **(E)** Example of a representative BG agar plate with growing *Bp* WT colonies recovered from infected mouse lung homogenates 7 days after infection. Magenta fluorescent colonies were readily distinguishable from white nonfluorescent colonies (indicated by arrows) and their proportion was counted. **(F)** Percentage of fluorescent colonies recovered from mouse lung and mLN homogenates at indicated days after inoculation by different mScarlet-producing strains. Data represent mean values from 3 independent colonization experiments.
(TIF)

S2 Fig *B*. *pertussis* producing enzymatically inactivated adenylate cyclase toxin (*Bp* AC^–^) persists in lungs for 21 days. Mice were intranasally inoculated with 50 μl of bacterial suspension containing 8 x 10^5^ CFU of the indicated *B*. *pertussis* strains (mScarlet^+^) and lung homogenates were prepared and plated on BG agar at indicated times. Means with SD of CFU from one infection experiment is shown (n = 3 mice/group).
(TIF)

S3 Fig Adenylate cyclase and pertussis toxins synergize in promoting lung pathology. Mice were intranasally inoculated with 8 x 10^5^ CFU of the indicated *B*. *pertussis* strains (mScarlet^+^). On day 5 and 14 mice were sacrificed and lungs were processed for histological examination. At the dissection, the *Bp* WT-infected lung tissue was fragile, whereas the *Bp* AC^−^ and *Bp* PT^−^-infected and control (sterile PBS-treated) lungs were compact and firm on day 5. In contrast, all infected lungs were enlarged and fragile on day 14. H&E-stained paraffin-embedded longitudinal lung sections were prepared as described in Materials and Methods. The experiment was performed twice independently (n = 3 mice/group) and representative images from one experiment are shown. Histopathology analysis revealed that the bronchopneumonia observed by day 5 in *Bp* WT, AC^−^ and PT^–^-infected mice was characterized by peribronchial interstitial edema with abnormal bronchial dilation, and by localized peribronchial, perivascular and alveolar inflammation, as compared to the healthy mouse lungs with normal bronchial and alveolar structure. The *Bp* WT strain provoked the highest degree of inflammatory damage. The low-colonizing double mutant *Bp* AC^–^PT^–^-infected mice showed only mild alveolar inflammation by day 5, characterized by sparse interstitial infiltrates. Pseudostratified bronchial epithelium was not damaged in any group by day 5. The highest pathology was observed in *Bp* WT-infected mice on day 14. The massive inflammation affected a large portion of the parenchyma, causing a lobar pneumonia. Bronchi were dilated and scarred, surrounded by diffuse peribronchial inflammation. The *Bp* AC^−^ strain elicited a more pronounced bronchopneumonia on day 14 compared to day 5 and compared to the *Bp* PT^−^ strain on day 14. The scale bar represents 200 μm.
(TIF)

S4 Fig mLN colonization by *Bp* PT^−^ peaks on day 14. Mice were intranasally inoculated with 50 μl of bacterial suspension containing 8 x 10^5^ CFU of the indicated *B*. *pertussis* strains (mScarlet^+^) and mLN homogenates were prepared and plated on BG agar at indicated times. Means with SD of CFU from one infection experiment is shown (n = 3 mice/group).
(TIF)

S5 Fig Persistence of mScarlet protein production by *B*. *pertussis* in the absence of antibiotic selection in the course of *in vivo* infection. Mice were intranasally inoculated with 50 μl of bacterial suspension containing 8 x 10^5^ CFU of *Bp* PT^−^ strain (mScarlet^+^) and on days 5 (A) and 14 (B) after infection, mLNs were collected, fixed in 4% PFA and snap-frozen. 10 μm cryosections were stained with anti-*B*. *pertussis* rabbit serum followed by goat anti-rabbit AF647-conjugated F(ab’2) IgG secondary staining (green); nuclei were counterstained with DAPI (blue), mScarlet fluorescent *Bp* PT^−^ is shown in magenta. The left panels show lower magnification images (scale bar 20 μm), right panels high magnification images (scale bar 10 μm). Top panels represent anti-*Bp* serum staining, bottom panels a negative control without anti-*Bp* serum, only goat anti-rabbit AF647-conjugated F(ab’2) IgG was added. Within each subpanel, an overlay image on the left is complemented by a single channel image (top, anti-*Bp* serum staining and bottom, *Bp* mScarlet) on the right side. The mScarlet-producing bacteria on mLN sections render brightly fluorescent intact coccobacilli, whereas immunofluorescent staining with anti-*B*. *pertussis* serum colocalized with the mScarlet signal but stained on the top of intact coccobacilli also the antigens from disintegrated bacteria. Two mLNs were analyzed at each indicated time point. Minimal loss of mScarlet fluorescence *in vivo* over the 14 days of infection was observed as compared to total anti-*B*. *pertussis* staining.
(TIF)

S6 Fig Stitched image of an mLN section from *Bp* WT-intected mouse on day 5. Higher resolution image of entire mLN section shown in Fig 4. Stitched image was acquired at 40x magnification by a IX83 fully-motorized and automated inverted fluorescence microscope (Olympus). Bacteria are encircled by yellow dotted lines. T cells, B cells, nuclei and bacteria are rendered in white, green, blue and magenta colors, respectively.
(TIF)

S7 Fig Stitched image of an mLN section from *Bp* PT^–^-intected mouse on day 5. Higher resolution image of entire mLN section shown in Fig 4. Stitched image was acquired at 40x magnification by a IX83 fully-motorized and automated inverted fluorescence microscope (Olympus). Bacteria are encircled by yellow dotted lines. T cells, B cells, nuclei and bacteria are rendered in white, green, blue and magenta colors, respectively.
(TIF)

S8 Fig Stitched image of an mLN section from *Bp* WT-intected mouse on day 14. Higher resolution image of entire mLN section shown in Fig 4. Stitched image was acquired at 40x magnification by a IX83 fully-motorized and automated inverted fluorescence microscope (Olympus). Bacteria are encircled by yellow dotted lines. T cells, B cells, nuclei and bacteria are rendered in white, green, blue and magenta colors, respectively.
(TIF)

S9 Fig Stitched image of an mLN section from *Bp* PT^–^-intected mouse on day 14. Higher resolution image of entire mLN section shown in Fig 4. Stitched image was acquired at 40x magnification by a IX83 fully-motorized and automated inverted fluorescence microscope (Olympus). Bacteria are encircled by yellow dotted lines. T cells, B cells, nuclei and bacteria are rendered in white, green, blue and magenta colors, respectively.
(TIF)

S10 Fig *Bp* PT^−^ first enters the T-cell zone of mLNs and relocates into the B-cell zone by day 14 of infection. Additional images to document observations made in Fig 4. Immunofluorescence microscopy of cryosections of mLNs of infected mice on day 5 (upper panel) and day 14 (lower panel). mLNs were fixed with 4% PFA, snap frozen and 10 μm longitudinal cryosections were first labeled with rat anti-mouse CD45R (B220), followed by goat anti-rat Alexa Fluor 488 secondary antibody conjugate and next Alexa Fluor 647 rat anti-mouse CD3 antibody conjugate was added. Nuclei were labeled with DAPI. Scale bar 20 μm. Bacteria are indicated by magenta arrows. T cells, B cells, nuclei and bacteria are rendered in white, green, blue and magenta colors, respectively. Images were acquired using a Leica TCS SPE confocal microscope.
(TIFF)

S11 Fig PT-deficient *B*. *pertussis* bacteria proliferate in mLNs. Additional images in support of observations shown in Fig 5. **(A)** Confocal microscopy images showing the clustering of *Bp* PT^−^ bacteria in the mLNs on day 14. Tissues were processed as described in the legend to Fig 5A. Nuclei and bacteria are in blue and magenta, respectively. Scale bar 10 μm. **(B)** Confocal microscopy images of mLN cryosections on day 5 and day 14 after intranasal challenge of mice with a 1:1 mixture of *Bp* PT^−^ strains producing mScarlet and GFP fluorescent proteins. At the indicated time points, the mLNs were processed as described in the legend to Fig 5C. The mScarlet and GFP-producing bacteria are rendered in magenta and green, respectively. Scale bar 10 μm.
(TIF)

S12 Fig Gating strategy used for identification of cell populations in mediastinal lymph nodes of *Bp*-infected mice. A representative gating strategy is shown on a sample of a mLN suspension from a *Bp* WT-infected mouse on day 5. mLN cell suspensions were prepared by enzymatic disruption. Cells were stained with a panel of fluorescently-labeled antibodies targeting cell-surface antigens (CD3-V500, CD19-A700, Ly6G-PerCP, Ly6C-AF488, CD11c-eF450, MHC-II-APC, CD11b-PE-Cy7; see S1 Table for details). Only single cell events from stable flow rate over time were selected, followed by dead cell elimination using a Fixable Viability Dye (FVD) eFluor 780 live/dead stain. After the gating of neutrophils (CD11b^+^Ly6G^+^), plasmacytoid DCs (pDCs) were identified as Ly6C^high^ CD11b^–^ and further confirmed to be CD11c^+^MHC-II^low^. T-cells were separated using a CD3 marker. Dendritic cells (DCs) were identified on the basis of their autofluorescence in V500 channel together with CD11c and MHC-II positivity. Conventional DCs (cDCs) were left after a population of monocyte-derived DCs (moDCs), highly expressing Ly6C and CD11b markers, was gated out. Conventional DCs were then separated into migratory and resident cDCs according to Sheng *et al*., who showed that the MHC-II^high^ CD11c^int^ population had a phenotype of migratory cells, confirmed by their high expression of CCR7 and ability to be labeled by intranasally-applied CFSE [107]. Both migratory and resident cDCs were further separated on the basis of their CD11b expression into CD11b^low^ cDC1 (which typically express CD8 in lymphoid organs and CD103 in non-lymphoid organs) and CD11b^+^ cDC2 (CD11b^+^ DCs). Non-T and non-DC cells were further gated into MHC-II^+^CD19^+^ B-cells, SSC^high^ CD11b^+^ eosinophils and other SSC^low^CD11b^+^ myeloid cell populations defined as monocytes/macrophages (Mono/Mfs). The remaining gate contained some NK cells and erythrocytes.
(TIF)

S13 Fig Majority of *B*. *pertussis* isolated from mLNs were associated with cells. Mice were intranasally inoculated with 50 μl of bacterial suspension containing 8 x 10^5^ CFU of the indicated *B*. *pertussis* strains (mScarlet^+^). Pools of mLNs from individual mice were collected. mLN homogenates were prepared by pressing the mLNs through a 70 μm cell strainer in cold PBS. To separate the cell-associated and free bacteria in mLN suspensions, LN homogenates were kept on ice to prevent phagocytosis and bacteria were separated from mouse cells by low-speed centrifugation (300 × g, 5 min, 4°C). No antibiotic treatment was applied to kill extracellular bacteria. Pellets resuspended in PBS and supernatants were then plated separately on BG agar plates. A portion of CFU recovered from pellets was identified as cell-associated bacteria. **(A)** Proportion of cell-associated CFU from total CFU recovered from mLN homogenates, plotted as mean with SD. **(B)** Absolute numbers of cell-associated CFU, a point graph from individual mice. Lines indicate the means. Data shown in **(A, B)** show a result from one experiment (n = 5–6 mice /group in *Bp* WT and *Bp* PT^−^ groups and n = 4 mice / *Bp* AC^−^ group).
(TIF)

S14 Fig Identification of *B*. *pertussis* mScarlet^+^ events in cell suspensions of mediastinal lymph nodes. Mice were intranasally inoculated with 50 μl of bacterial suspension containing 8 x 10^5^ CFU of the indicated *B*. *pertussis* strains (mScarlet^+^), or with corresponding non-fluorescent strains. Cell suspensions were prepared from pools of mLNs collected on days 5 and 14 from 4 mice per each condition. mLN suspensions were pooled, stained with a panel of fluorescently-labeled antibodies and analyzed by flow cytometry. The indicated numbers represent mScarlet^+^ cells detected per 2 x 10^6^ events on day 5 and 14, except for day 14 of the mLNs of mice infected by *Bp* PT^–^, where the sample of analyzed cells was increased to 6 x 10^6^ events. mScarlet^+^ cells associated with *Bp* bacteria were detected using 585/15 nm emission filter and the gate was set using cellular suspension of mLNs from mice infected with the non-fluorescent control bacteria. *Bp*-associated cells were visualized in simple dot plots from down-sampled live mScarlet^+^ events (lower panels): MHC-II^+^ CD11c^+^ dendritic cells (DC, red dots), CD19^+^CD3^–^ B cells (blue dots) and CD11b^+^Ly6G^+^ neutrophils (green dots). Data from one representative experiment out of 3 (*Bp* WT) or 4 (*Bp* PT^–^) performed are shown.
(TIF)

S15 Fig Visualization of *Bp* PT^–^-infected CD11c^+^ dendritic cells on mLN cryosections on day 5. Additional images documenting the observations shown in Fig 8B. To visualize the infected dendritic cells on day 5, cryosections of mLNs from mice infected with *Bp* PT^−^ mScarlet were stained by a biotin-conjugated CD11c monoclonal antibody (clone HL3), detected by AF488-conjugated streptavidin. Dendritic cells, nuclei and bacteria are rendered in green, blue, and magenta colors, respectively. Scale bar = 10 μm.
(TIF)

S16 Fig Visualization of *Bp* PT^–^-infected neutrophils on mLN cryosections from day 14. Additional images documenting the observations shown in Fig 8C. Neutrophils on cryosections of mLNs from *Bp* PT^–^-infected mice on day 14 were stained with biotin-conjugated rat Ly6G antibody followed by AF488-conjugated streptavidin. Orthogonal views of *Bp* PT^−^ mScarlet-infected neutrophils clearly show intracellular localization of bacteria in neutrophils. Z-stack images were acquired using a confocal microscope. Neutrophils, nuclei and bacteria are rendered in green, blue, and magenta colors, respectively. Scale bar 10 μm.
(TIF)

S17 Fig Cytokine secretion by *B*. *pertussis* antigen-restimulated splenocytes from mice on days 5 and 14 after infection. Mice were intranasally inoculated with 50 μl of bacterial suspension containing 8 x 10^5^ CFU of the indicated *B*. *pertussis* strains (mScarlet^+^) and on day 5 and 14 spleens were collected. Splenocytes were isolated as previously described [11] and restimulated for 48 h by heat-killed *B*. *pertussis* WT (Tohama I), or by PBS and PMA/ionomycin (eBiosciences) used as negative and positive controls, respectively. IFN-γ, IL-17A, IL-2, IL-6, IL-10, TNF-α and IL-12p70 concentrations were determined in the culture supernatants of splenocytes using a custom-made ProcartaPlex cytokine bead assay (ThermoFisher Scientific, USA) on a Bio-Plex 200 instrument (Bio-Rad, USA). The analysis was done once (n = 3 mice/group). Data are represented as means with SD. Circles represent values from individual mice.
(TIF)
