# Supplementary figures and images for: Pertussis toxin suppresses dendritic cell-mediated delivery of B. pertussis into lung-draining lymph nodes
Source: PLoS Pathog. 2022 Jun 6;18(6):e1010577. doi: 10.1371/journal.ppat.1010577 (PMC9216613; doi:10.1371/journal.ppat.1010577)

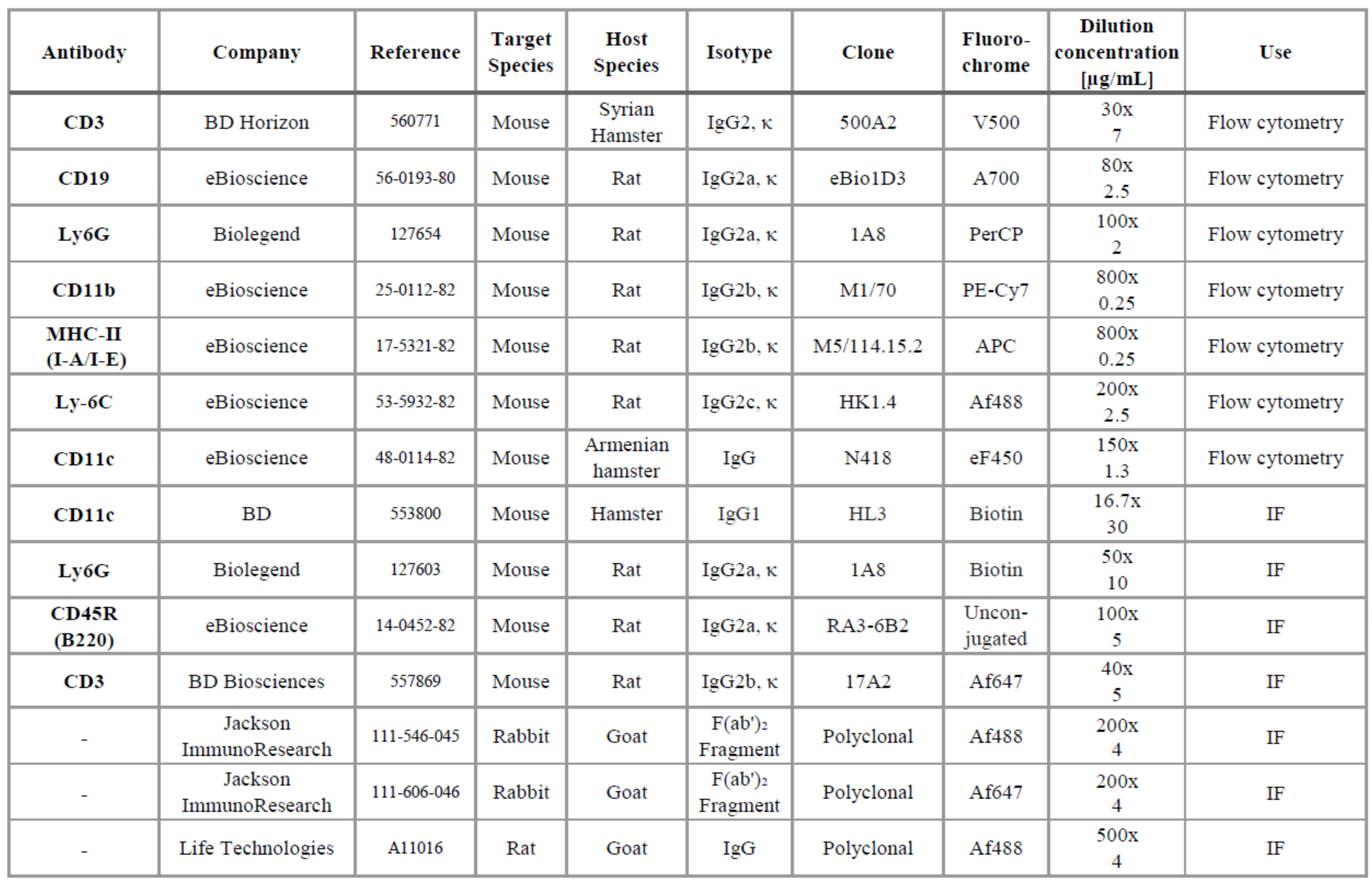

Supplement: S1 Table — IF (immunofluorescence), A700 (Alexa Fluor), PerCP (Peridinin-chlorophyll-protein Complex), PE-Cy7 (Phycoerythrin-Cyanine7), APC (Allophycocyanin), Af488 (Alexa Fluor), eF450 (eFluor), APC-eF780 (Allophycocyanin-eFluor), FITC (Fluorescein Isothiocyanate). (TIF) [file ppat.1010577.s002.tif]

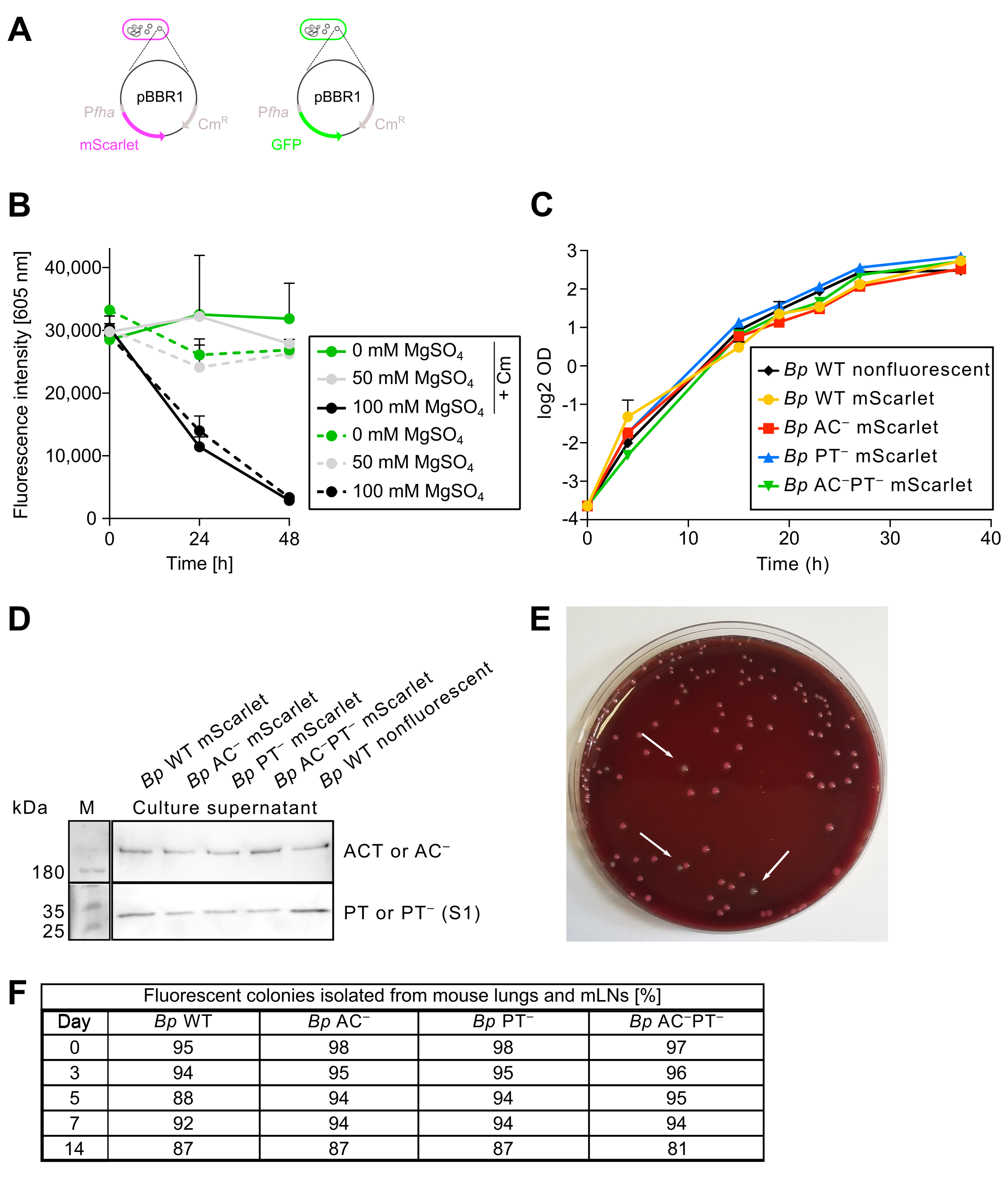

Supplement: S1 Fig — (A) Scheme of the pBBR1-derived plasmids for mScarlet and sfGFP production under the Bvg A/S-regulated Pfha promoter control. CmR chloramphenicol resistance gene. (B) Stability of mScarlet fluorescent protein production in liquid culture. Bacteria were grown at 37°C in 3 ml of modified SS medium supplemented with 50 or 100 mM MgSO4, and in the presence or absence of 10 μg/ml chloramphenicol for 24 hours. Afterwards the culture was diluted to OD600 = 0.2 in the same medium and the bacteria were grown for another 24 hours. Fluorescence intensity at 605 nm was determined every 24 h in cultures diluted to OD = 0.2. (C) Growth curves of the used B. pertussis strains. Bacteria were grown in liquid cultures in 10 ml of modified SS medium without antibiotics at 37°C. OD600nm was measured in appropriately diluted cultures at indicated time points. Means +/- SD from one experiment performed in duplicate are shown. Data are representative of three independent experiments. (D) Toxin or toxoid production by the used strains. Bacteria were grown without antibiotics in SS medium containing 2 mM CaCl2 to facilitate ACT secretion [106]. Culture supernatants were collected at OD = 1 by centrifugation and probed for ACT and PT antigen by Western blotting. (E) Example of a representative BG agar plate with growing Bp WT colonies recovered from infected mouse lung homogenates 7 days after infection. Magenta fluorescent colonies were readily distinguishable from white nonfluorescent colonies (indicated by arrows) and their proportion was counted. (F) Percentage of fluorescent colonies recovered from mouse lung and mLN homogenates at indicated days after inoculation by different mScarlet-producing strains. Data represent mean values from 3 independent colonization experiments. (TIF) [file ppat.1010577.s003.tif]

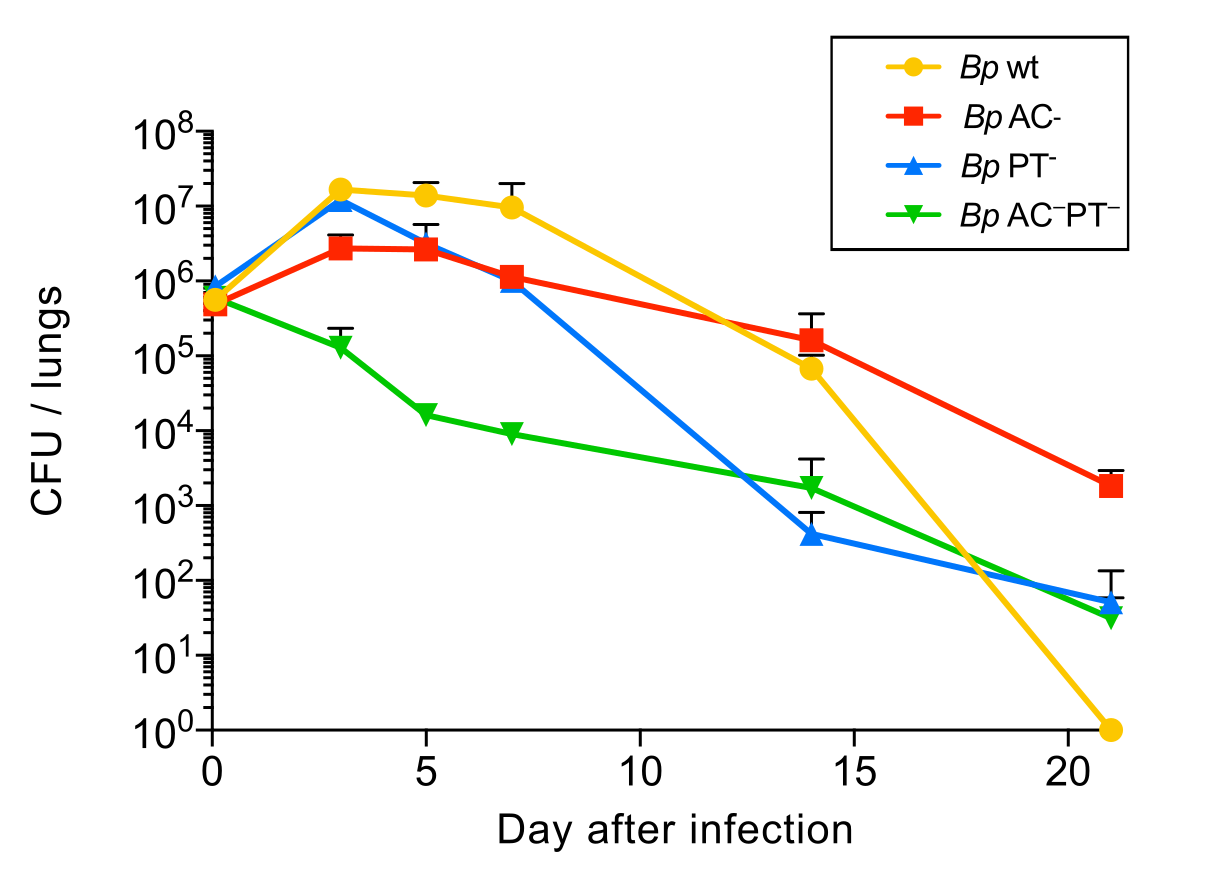

Supplement: S2 Fig — Mice were intranasally inoculated with 50 μl of bacterial suspension containing 8 x 10^5^ CFU of the indicated B. pertussis strains (mScarlet+) and lung homogenates were prepared and plated on BG agar at indicated times. Means with SD of CFU from one infection experiment is shown (n = 3 mice/group). (TIF) [file ppat.1010577.s004.tif]

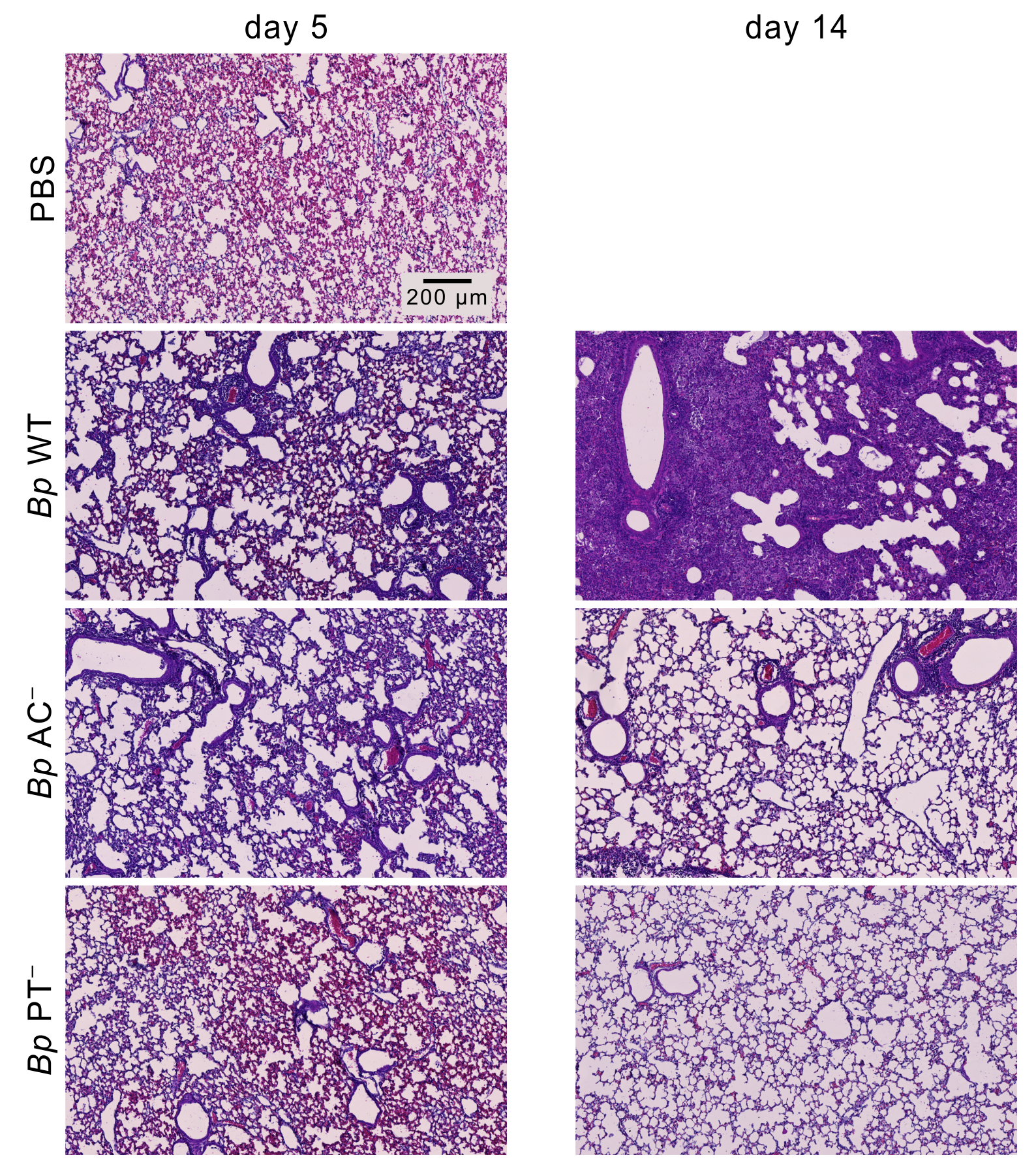

Supplement: S3 Fig — Mice were intranasally inoculated with 8 x 10^5^ CFU of the indicated B. pertussis strains (mScarlet+). On day 5 and 14 mice were sacrificed and lungs were processed for histological examination. At the dissection, the Bp WT-infected lung tissue was fragile, whereas the Bp AC− and Bp PT−-infected and control (sterile PBS-treated) lungs were compact and firm on day 5. In contrast, all infected lungs were enlarged and fragile on day 14. H&E-stained paraffin-embedded longitudinal lung sections were prepared as described in Materials and Methods. The experiment was performed twice independently (n = 3 mice/group) and representative images from one experiment are shown. Histopathology analysis revealed that the bronchopneumonia observed by day 5 in Bp WT, AC− and PT–-infected mice was characterized by peribronchial interstitial edema with abnormal bronchial dilation, and by localized peribronchial, perivascular and alveolar inflammation, as compared to the healthy mouse lungs with normal bronchial and alveolar structure. The Bp WT strain provoked the highest degree of inflammatory damage. The low-colonizing double mutant Bp AC–PT–-infected mice showed only mild alveolar inflammation by day 5, characterized by sparse interstitial infiltrates. Pseudostratified bronchial epithelium was not damaged in any group by day 5. The highest pathology was observed in Bp WT-infected mice on day 14. The massive inflammation affected a large portion of the parenchyma, causing a lobar pneumonia. Bronchi were dilated and scarred, surrounded by diffuse peribronchial inflammation. The Bp AC− strain elicited a more pronounced bronchopneumonia on day 14 compared to day 5 and compared to the Bp PT− strain on day 14. The scale bar represents 200 μm. (TIF) [file ppat.1010577.s005.tif]

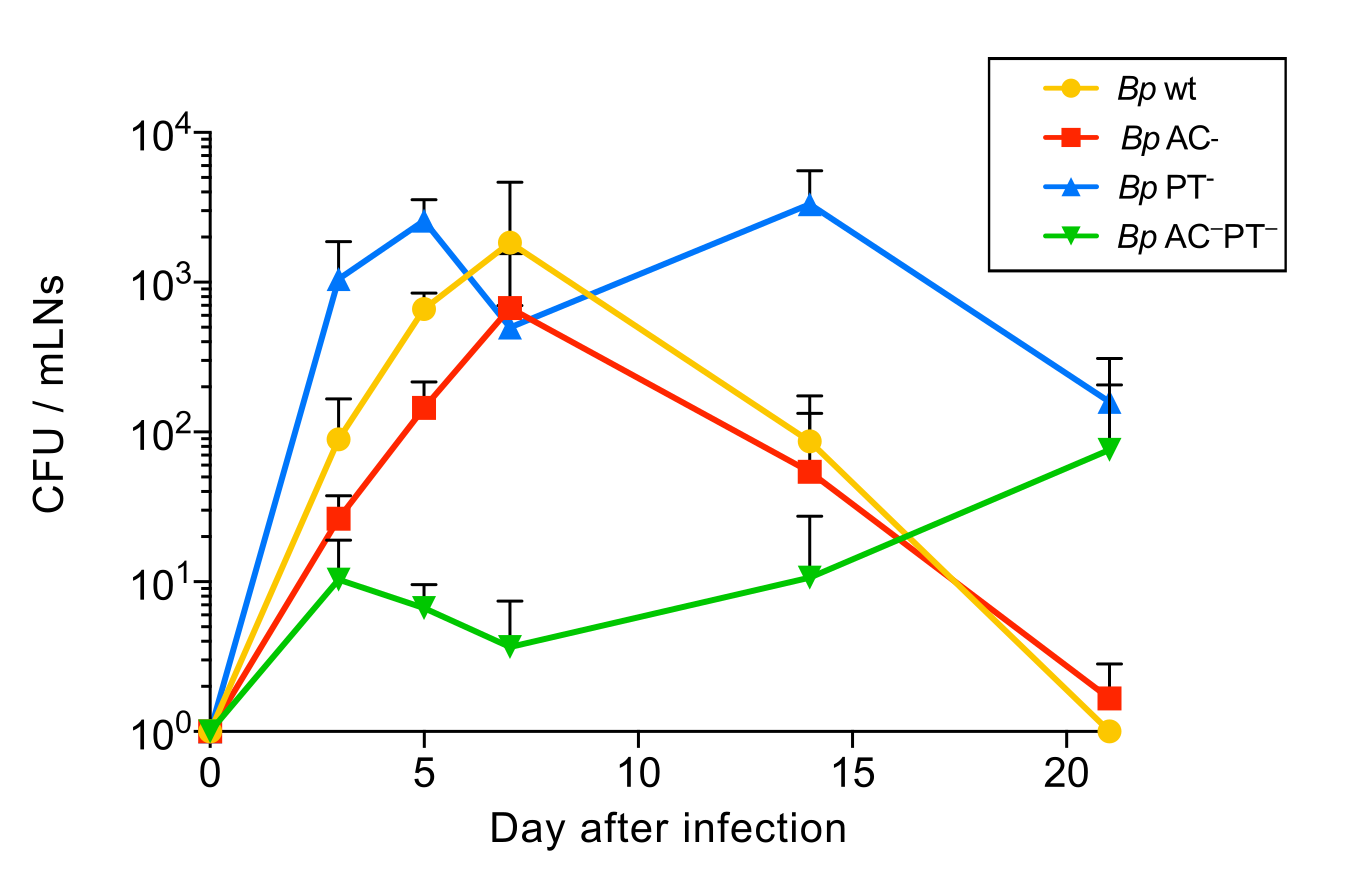

Supplement: S4 Fig — Mice were intranasally inoculated with 50 μl of bacterial suspension containing 8 x 10^5^ CFU of the indicated B. pertussis strains (mScarlet+) and mLN homogenates were prepared and plated on BG agar at indicated times. Means with SD of CFU from one infection experiment is shown (n = 3 mice/group). (TIF) [file ppat.1010577.s006.tif]

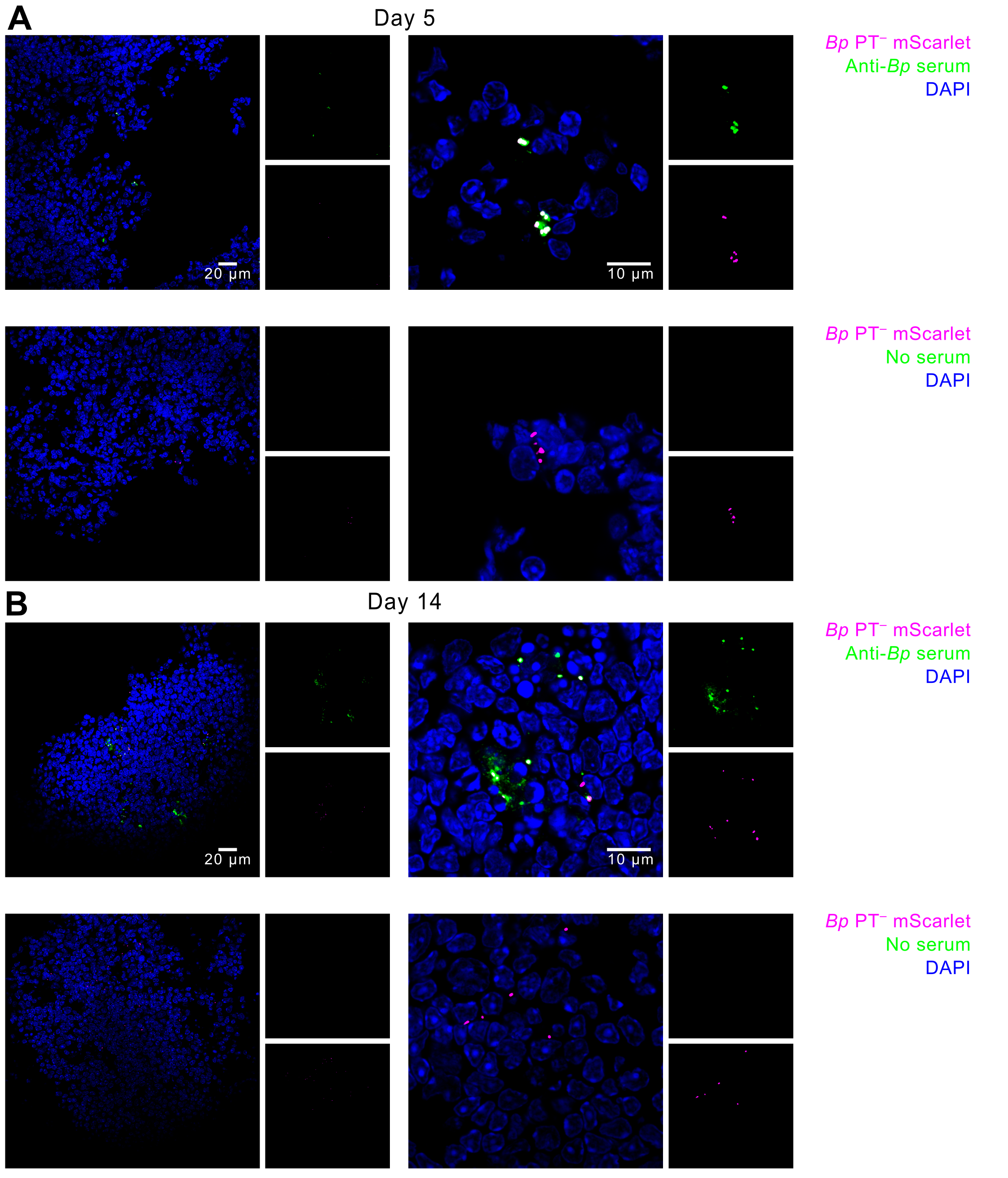

Supplement: S5 Fig — Mice were intranasally inoculated with 50 μl of bacterial suspension containing 8 x 10^5^ CFU of Bp PT− strain (mScarlet+) and on days 5 (A) and 14 (B) after infection, mLNs were collected, fixed in 4% PFA and snap-frozen. 10 μm cryosections were stained with anti-B. pertussis rabbit serum followed by goat anti-rabbit AF647-conjugated F(ab’2) IgG secondary staining (green); nuclei were counterstained with DAPI (blue), mScarlet fluorescent Bp PT− is shown in magenta. The left panels show lower magnification images (scale bar 20 μm), right panels high magnification images (scale bar 10 μm). Top panels represent anti-Bp serum staining, bottom panels a negative control without anti-Bp serum, only goat anti-rabbit AF647-conjugated F(ab’2) IgG was added. Within each subpanel, an overlay image on the left is complemented by a single channel image (top, anti-Bp serum staining and bottom, Bp mScarlet) on the right side. The mScarlet-producing bacteria on mLN sections render brightly fluorescent intact coccobacilli, whereas immunofluorescent staining with anti-B. pertussis serum colocalized with the mScarlet signal but stained on the top of intact coccobacilli also the antigens from disintegrated bacteria. Two mLNs were analyzed at each indicated time point. Minimal loss of mScarlet fluorescence in vivo over the 14 days of infection was observed as compared to total anti-B. pertussis staining. (TIF) [file ppat.1010577.s007.tif]

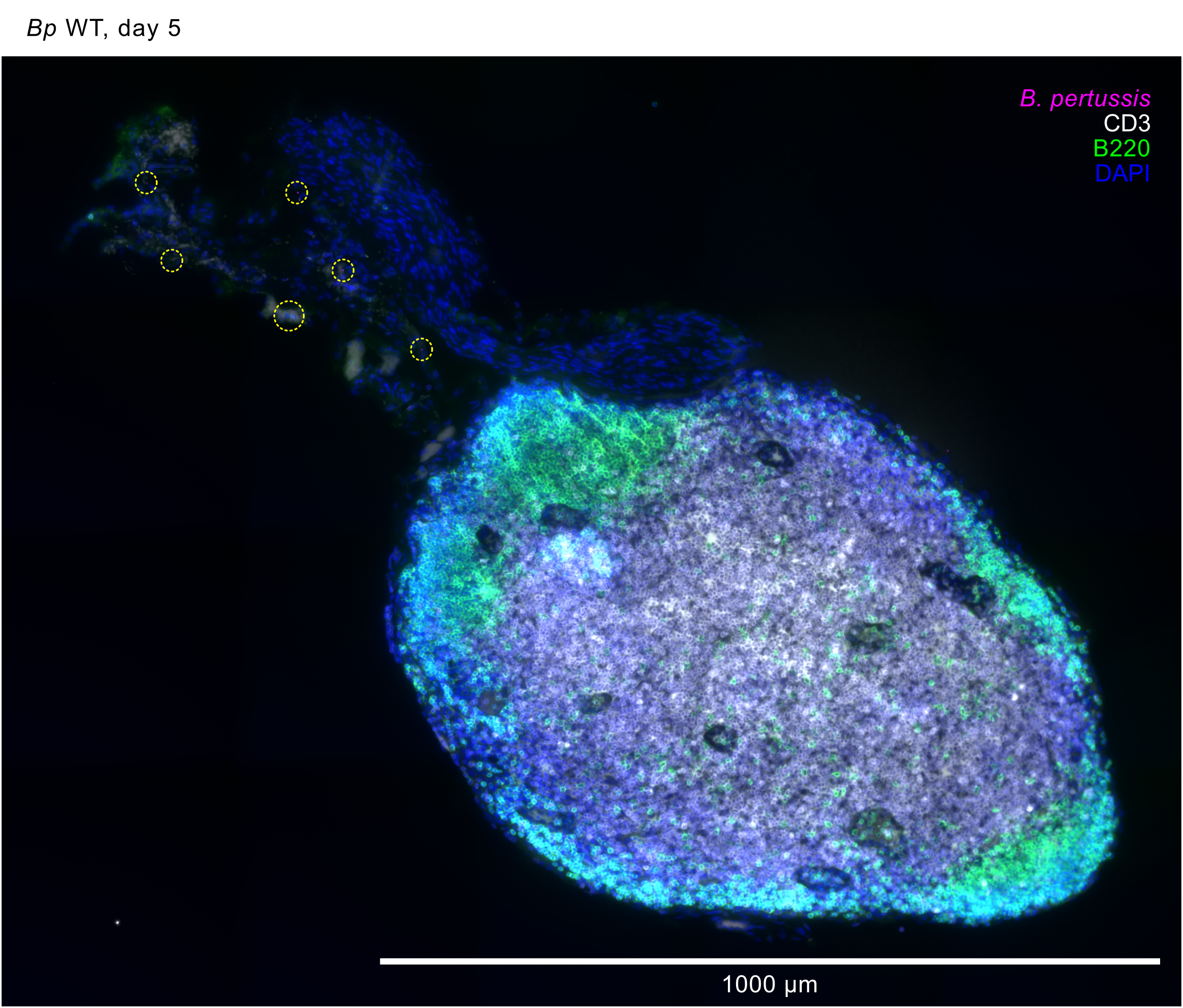

Supplement: S6 Fig — Higher resolution image of entire mLN section shown in Fig 4. Stitched image was acquired at 40x magnification by a IX83 fully-motorized and automated inverted fluorescence microscope (Olympus). Bacteria are encircled by yellow dotted lines. T cells, B cells, nuclei and bacteria are rendered in white, green, blue and magenta colors, respectively. (TIF) [file ppat.1010577.s008.tif]

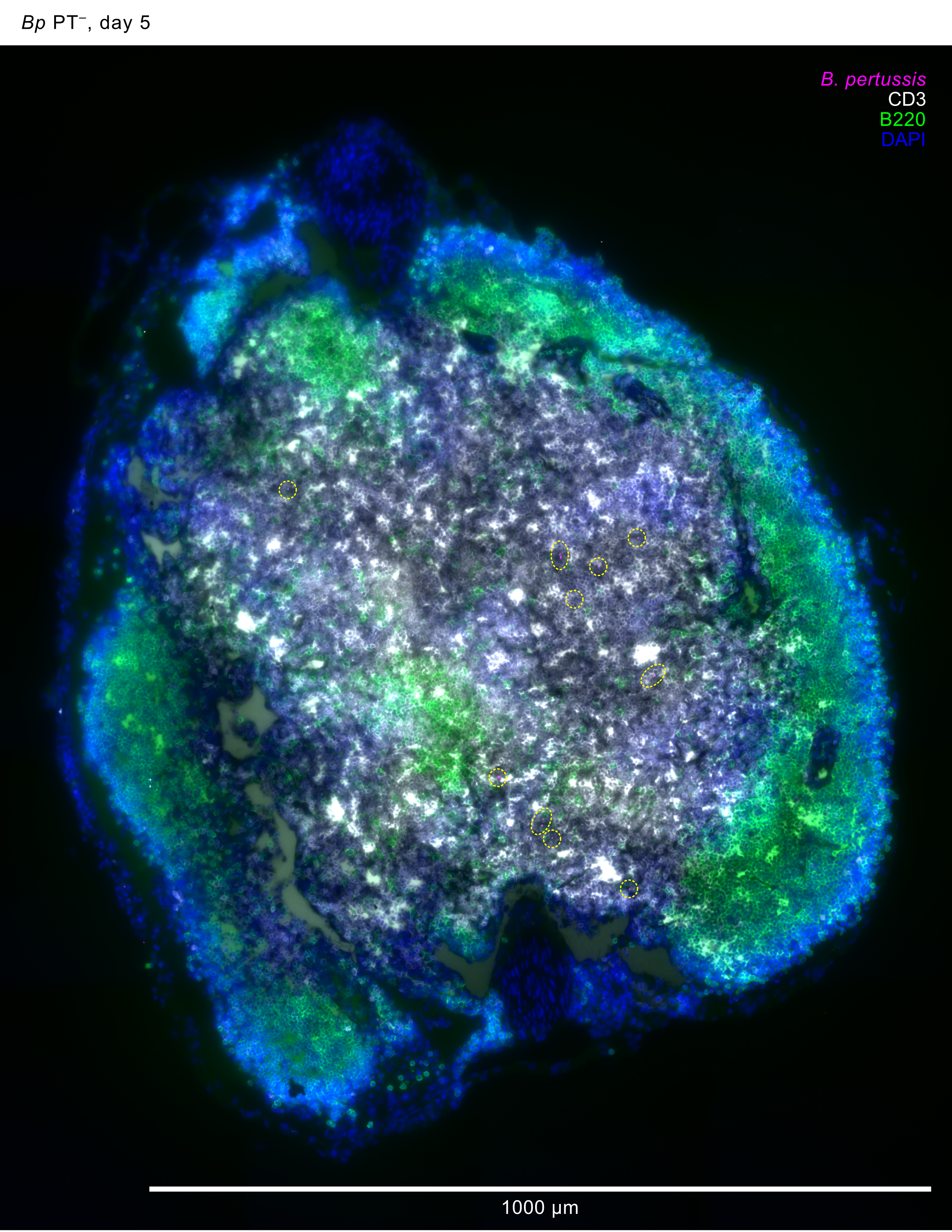

Supplement: S7 Fig — Higher resolution image of entire mLN section shown in Fig 4. Stitched image was acquired at 40x magnification by a IX83 fully-motorized and automated inverted fluorescence microscope (Olympus). Bacteria are encircled by yellow dotted lines. T cells, B cells, nuclei and bacteria are rendered in white, green, blue and magenta colors, respectively. (TIF) [file ppat.1010577.s009.tif]

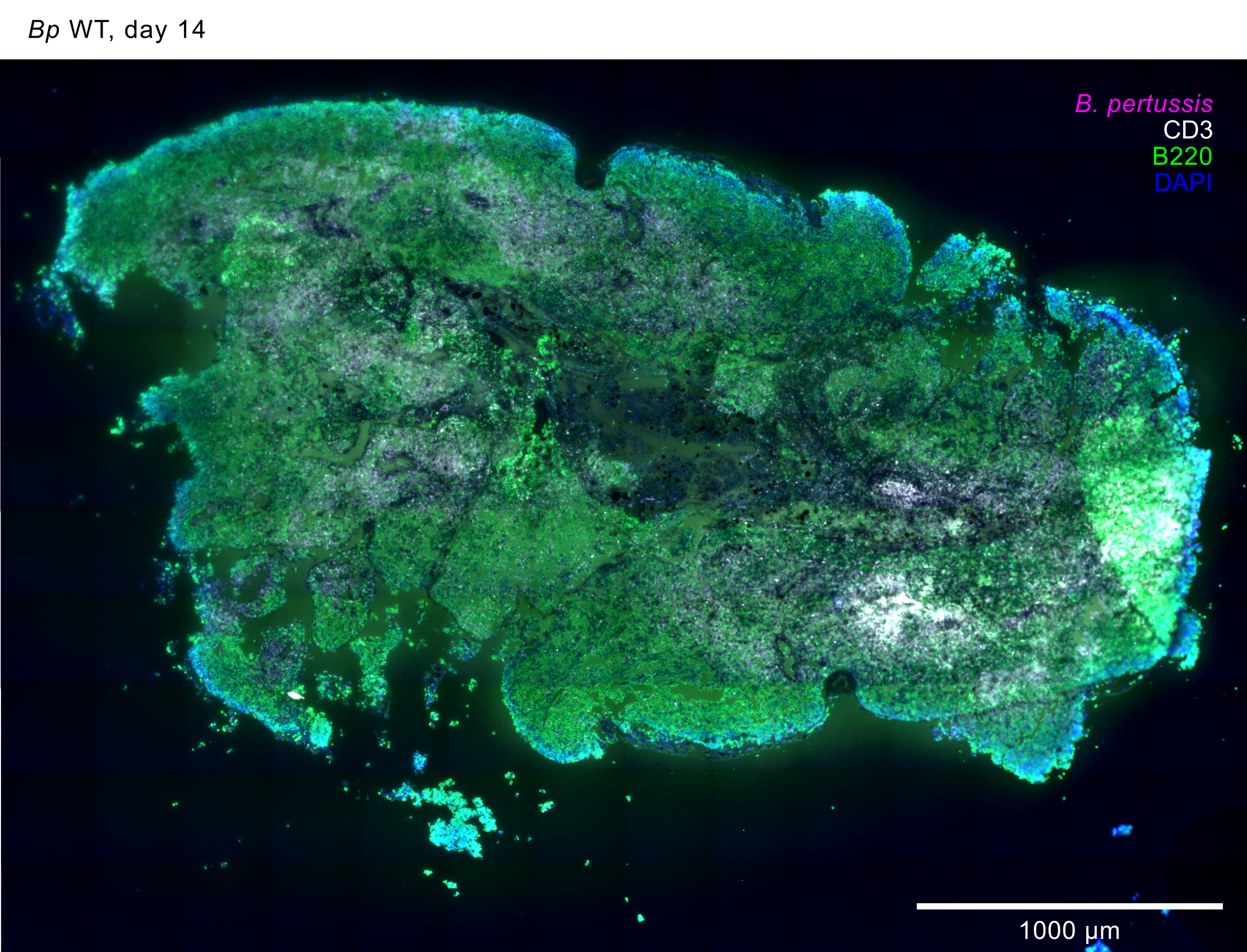

Supplement: S8 Fig — Higher resolution image of entire mLN section shown in Fig 4. Stitched image was acquired at 40x magnification by a IX83 fully-motorized and automated inverted fluorescence microscope (Olympus). Bacteria are encircled by yellow dotted lines. T cells, B cells, nuclei and bacteria are rendered in white, green, blue and magenta colors, respectively. (TIF) [file ppat.1010577.s010.tif]

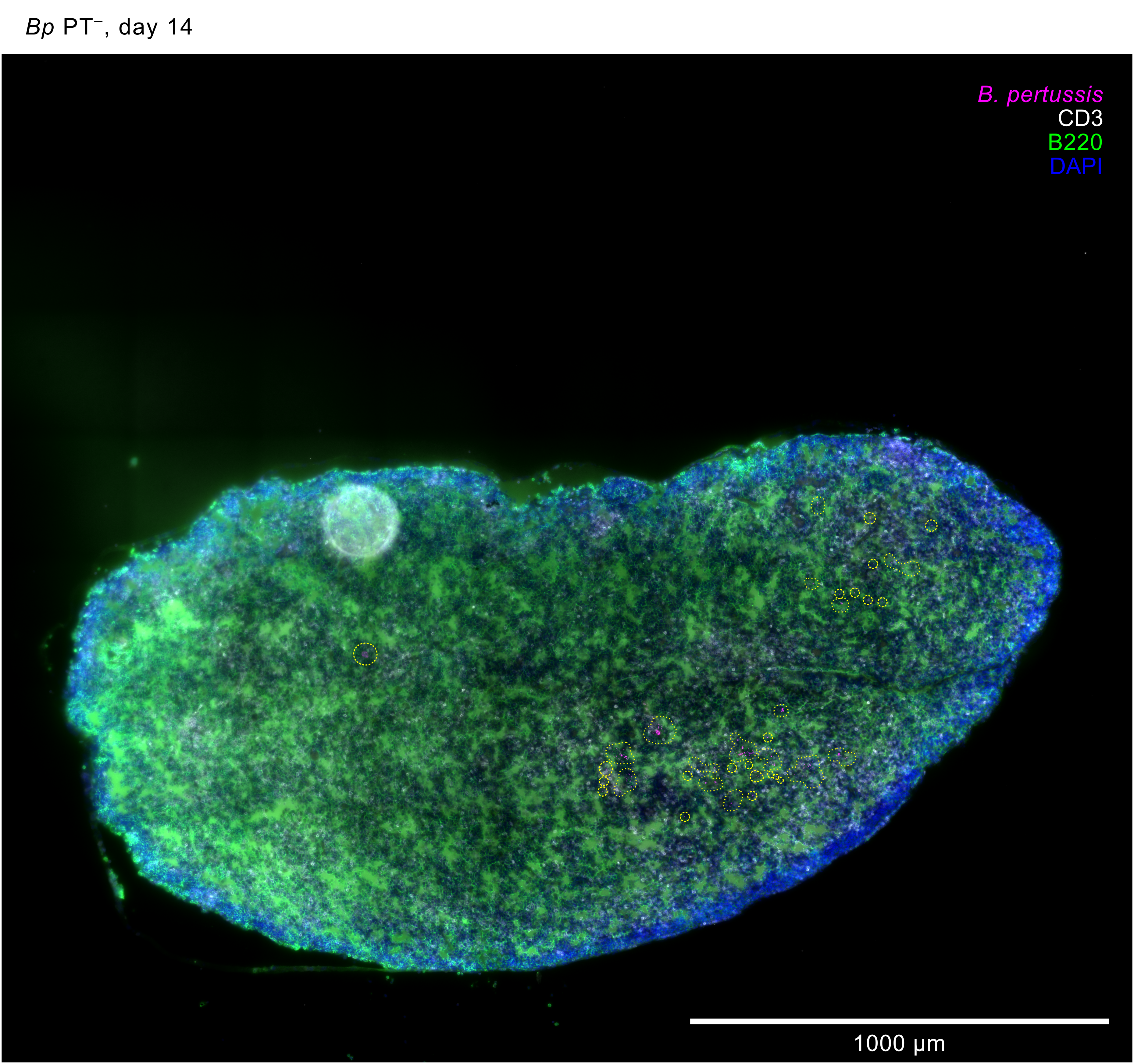

Supplement: S9 Fig — Higher resolution image of entire mLN section shown in Fig 4. Stitched image was acquired at 40x magnification by a IX83 fully-motorized and automated inverted fluorescence microscope (Olympus). Bacteria are encircled by yellow dotted lines. T cells, B cells, nuclei and bacteria are rendered in white, green, blue and magenta colors, respectively. (TIF) [file ppat.1010577.s011.tif]

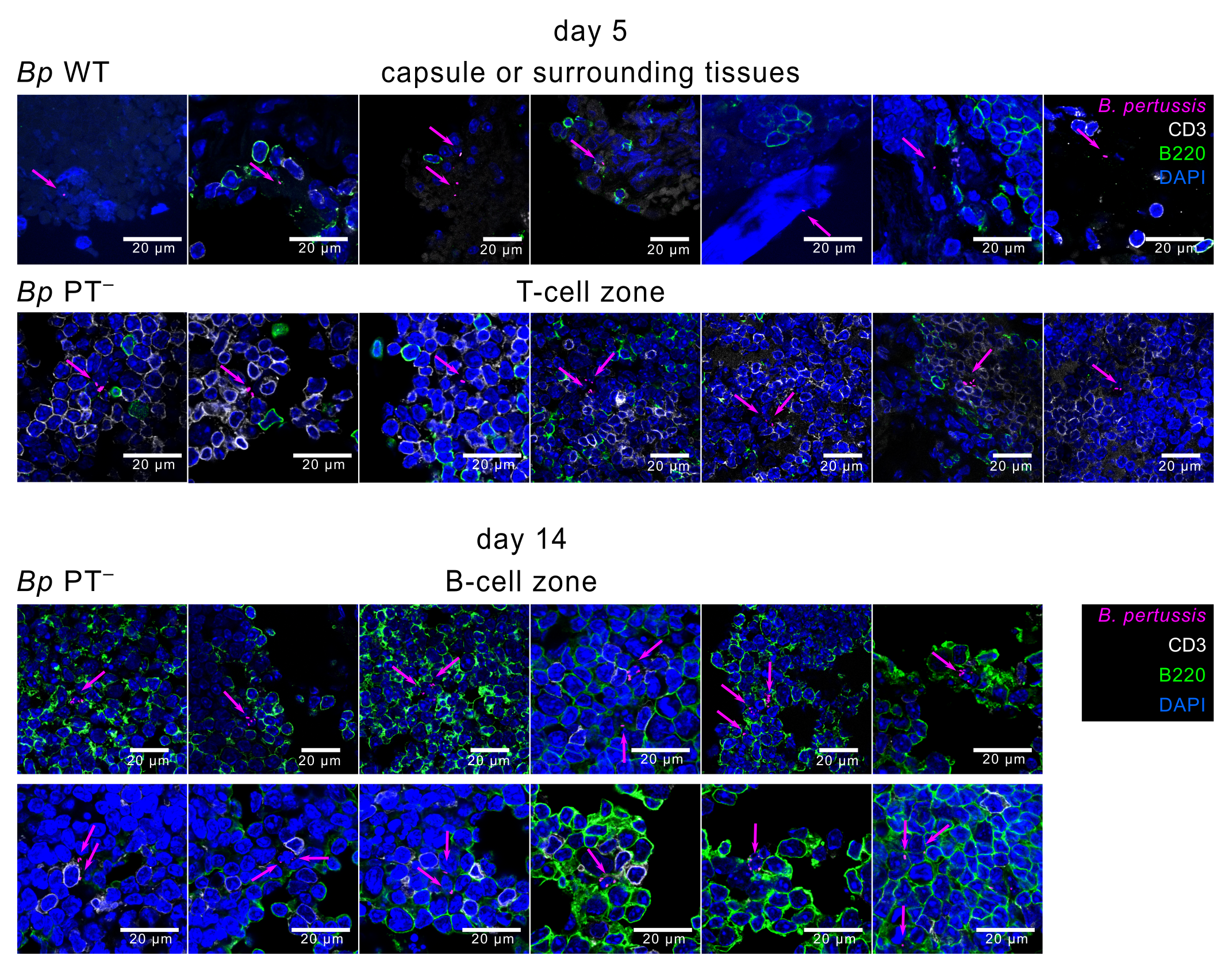

Supplement: S10 Fig — Additional images to document observations made in Fig 4. Immunofluorescence microscopy of cryosections of mLNs of infected mice on day 5 (upper panel) and day 14 (lower panel). mLNs were fixed with 4% PFA, snap frozen and 10 μm longitudinal cryosections were first labeled with rat anti-mouse CD45R (B220), followed by goat anti-rat Alexa Fluor 488 secondary antibody conjugate and next Alexa Fluor 647 rat anti-mouse CD3 antibody conjugate was added. Nuclei were labeled with DAPI. Scale bar 20 μm. Bacteria are indicated by magenta arrows. T cells, B cells, nuclei and bacteria are rendered in white, green, blue and magenta colors, respectively. Images were acquired using a Leica TCS SPE confocal microscope. (TIFF) [file ppat.1010577.s012.tiff]

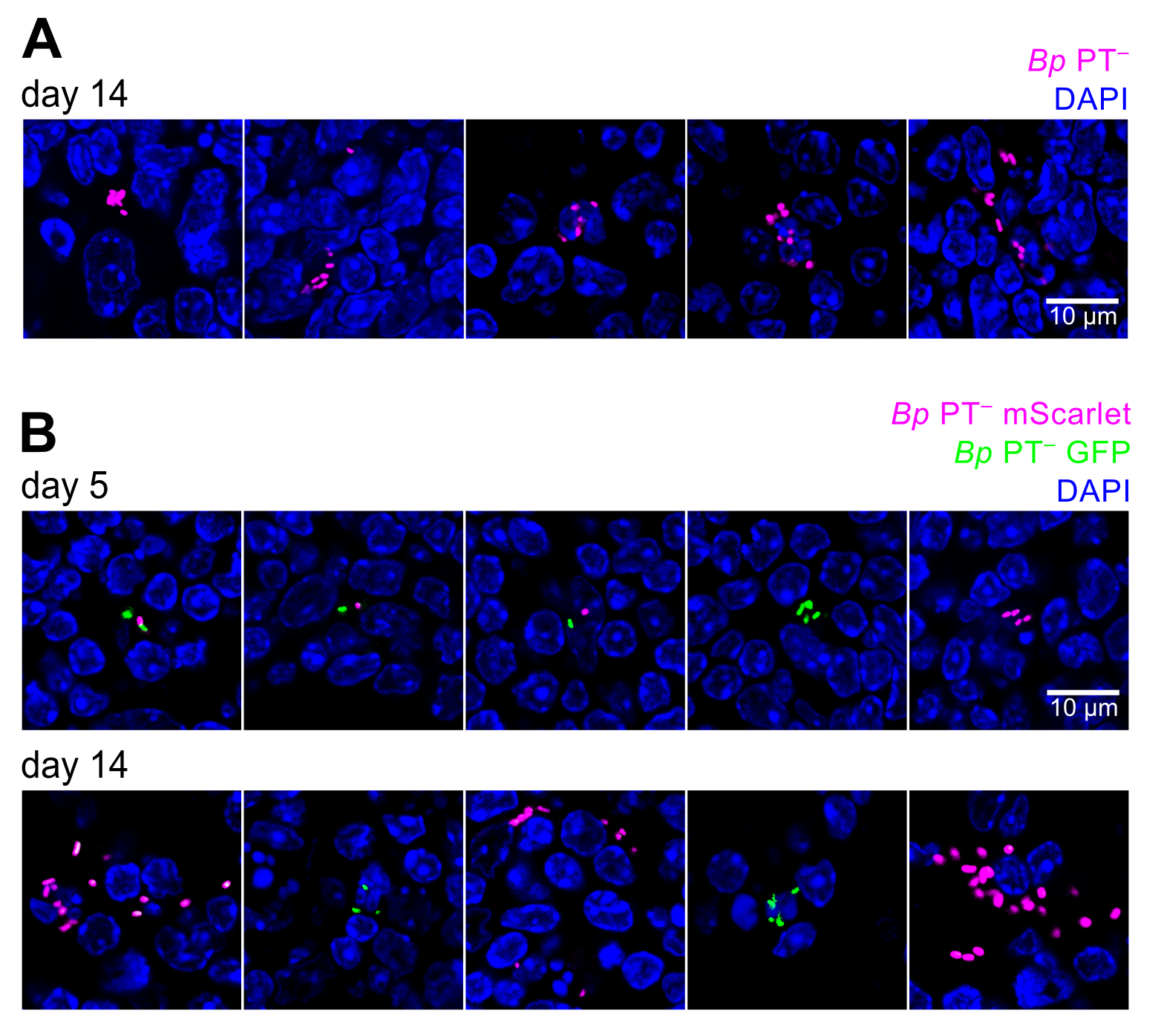

Supplement: S11 Fig — Additional images in support of observations shown in Fig 5. (A) Confocal microscopy images showing the clustering of Bp PT− bacteria in the mLNs on day 14. Tissues were processed as described in the legend to Fig 5A. Nuclei and bacteria are in blue and magenta, respectively. Scale bar 10 μm. (B) Confocal microscopy images of mLN cryosections on day 5 and day 14 after intranasal challenge of mice with a 1:1 mixture of Bp PT− strains producing mScarlet and GFP fluorescent proteins. At the indicated time points, the mLNs were processed as described in the legend to Fig 5C. The mScarlet and GFP-producing bacteria are rendered in magenta and green, respectively. Scale bar 10 μm. (TIF) [file ppat.1010577.s013.tif]

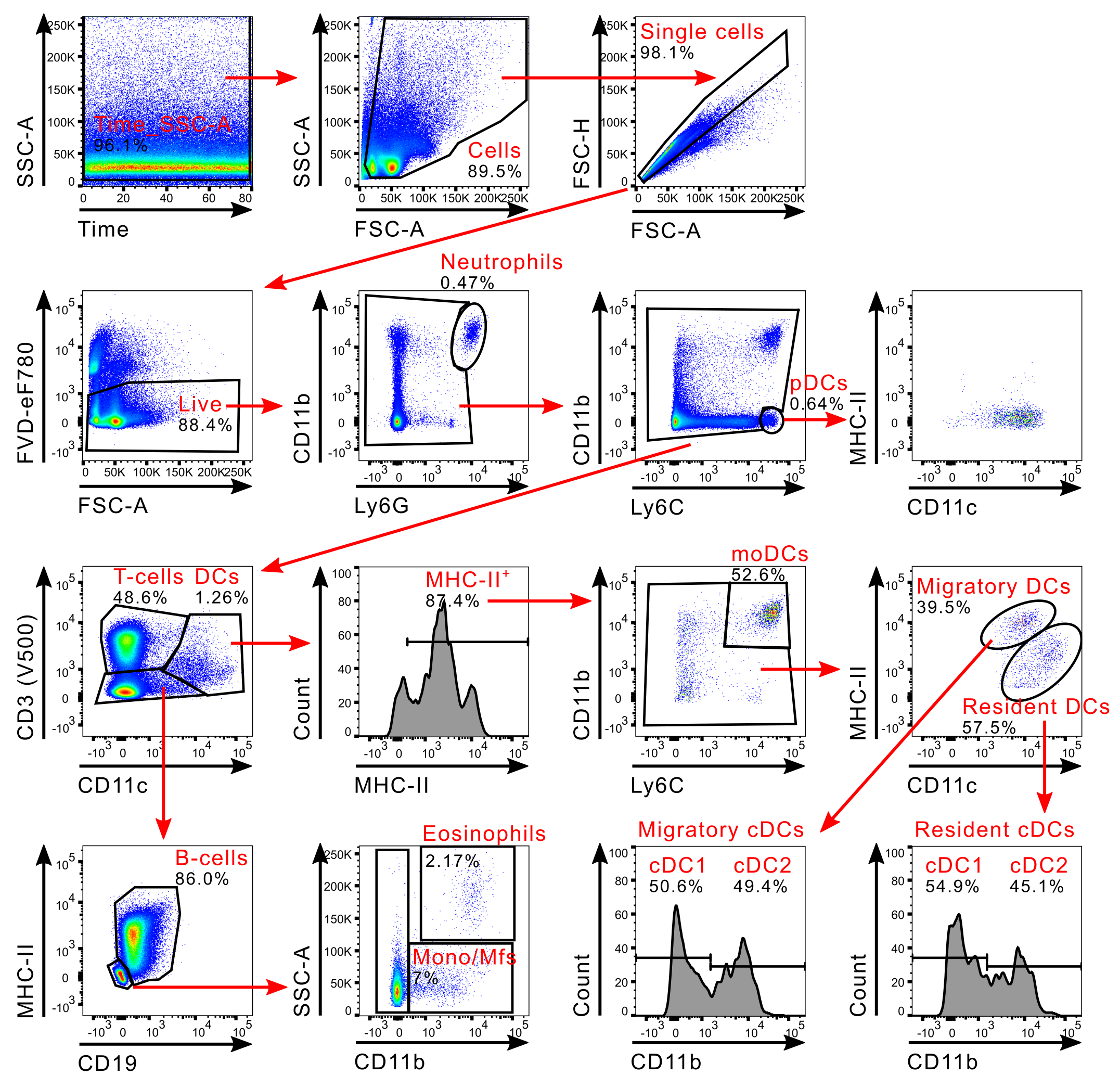

Supplement: S12 Fig — A representative gating strategy is shown on a sample of a mLN suspension from a Bp WT-infected mouse on day 5. mLN cell suspensions were prepared by enzymatic disruption. Cells were stained with a panel of fluorescently-labeled antibodies targeting cell-surface antigens (CD3-V500, CD19-A700, Ly6G-PerCP, Ly6C-AF488, CD11c-eF450, MHC-II-APC, CD11b-PE-Cy7; see S1 Table for details). Only single cell events from stable flow rate over time were selected, followed by dead cell elimination using a Fixable Viability Dye (FVD) eFluor 780 live/dead stain. After the gating of neutrophils (CD11b+Ly6G+), plasmacytoid DCs (pDCs) were identified as Ly6Chigh CD11b– and further confirmed to be CD11c+MHC-IIlow. T-cells were separated using a CD3 marker. Dendritic cells (DCs) were identified on the basis of their autofluorescence in V500 channel together with CD11c and MHC-II positivity. Conventional DCs (cDCs) were left after a population of monocyte-derived DCs (moDCs), highly expressing Ly6C and CD11b markers, was gated out. Conventional DCs were then separated into migratory and resident cDCs according to Sheng et al., who showed that the MHC-IIhigh CD11cint population had a phenotype of migratory cells, confirmed by their high expression of CCR7 and ability to be labeled by intranasally-applied CFSE [107]. Both migratory and resident cDCs were further separated on the basis of their CD11b expression into CD11blow cDC1 (which typically express CD8 in lymphoid organs and CD103 in non-lymphoid organs) and CD11b+ cDC2 (CD11b+ DCs). Non-T and non-DC cells were further gated into MHC-II+CD19+ B-cells, SSChigh CD11b+ eosinophils and other SSClowCD11b+ myeloid cell populations defined as monocytes/macrophages (Mono/Mfs). The remaining gate contained some NK cells and erythrocytes. (TIF) [file ppat.1010577.s014.tif]

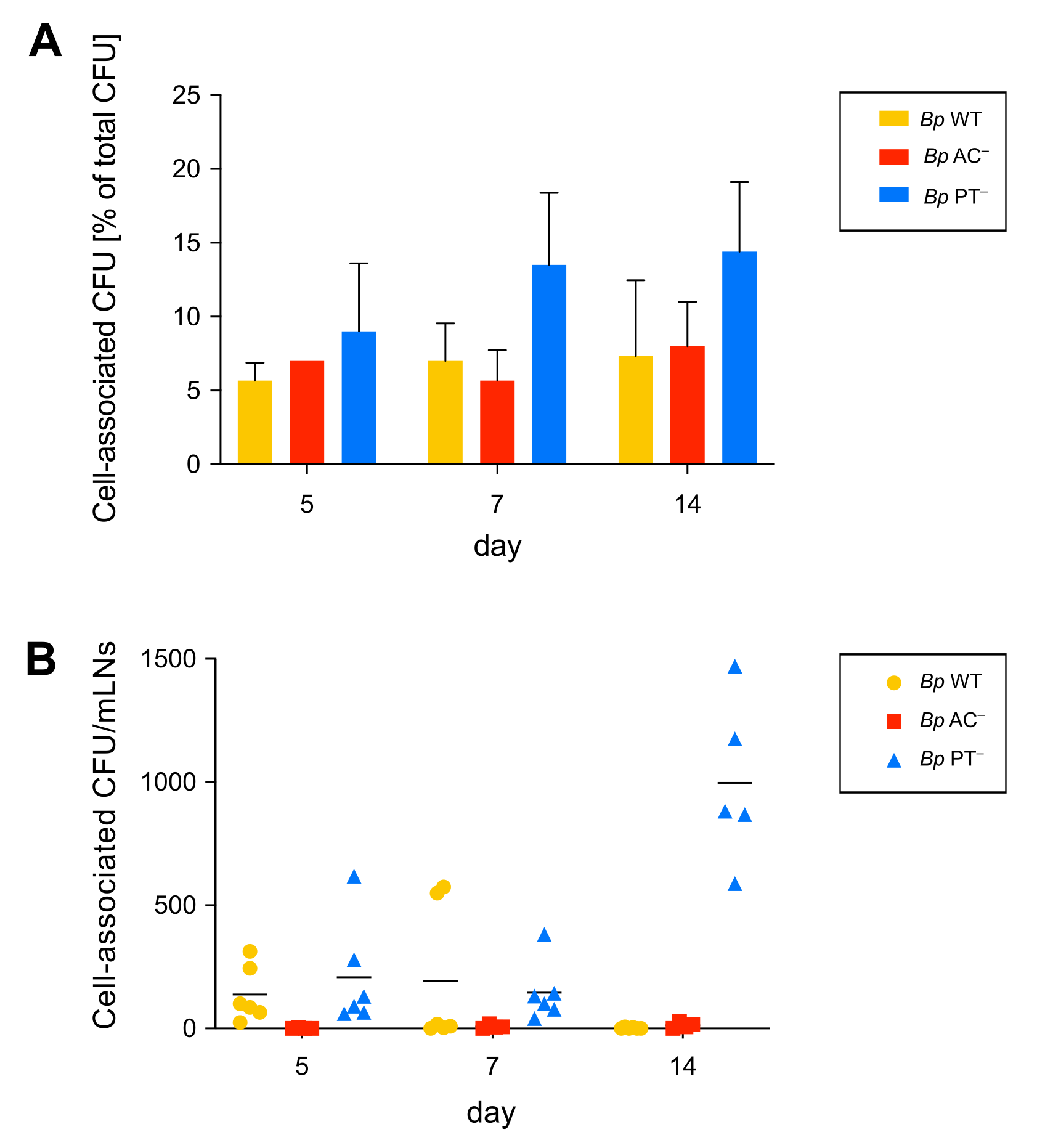

Supplement: S13 Fig — Mice were intranasally inoculated with 50 μl of bacterial suspension containing 8 x 10^5^ CFU of the indicated B. pertussis strains (mScarlet+). Pools of mLNs from individual mice were collected. mLN homogenates were prepared by pressing the mLNs through a 70 μm cell strainer in cold PBS. To separate the cell-associated and free bacteria in mLN suspensions, LN homogenates were kept on ice to prevent phagocytosis and bacteria were separated from mouse cells by low-speed centrifugation (300 × g, 5 min, 4°C). No antibiotic treatment was applied to kill extracellular bacteria. Pellets resuspended in PBS and supernatants were then plated separately on BG agar plates. A portion of CFU recovered from pellets was identified as cell-associated bacteria. (A) Proportion of cell-associated CFU from total CFU recovered from mLN homogenates, plotted as mean with SD. (B) Absolute numbers of cell-associated CFU, a point graph from individual mice. Lines indicate the means. Data shown in (A, B) show a result from one experiment (n = 5–6 mice /group in Bp WT and Bp PT− groups and n = 4 mice / Bp AC− group). (TIF) [file ppat.1010577.s015.tif]

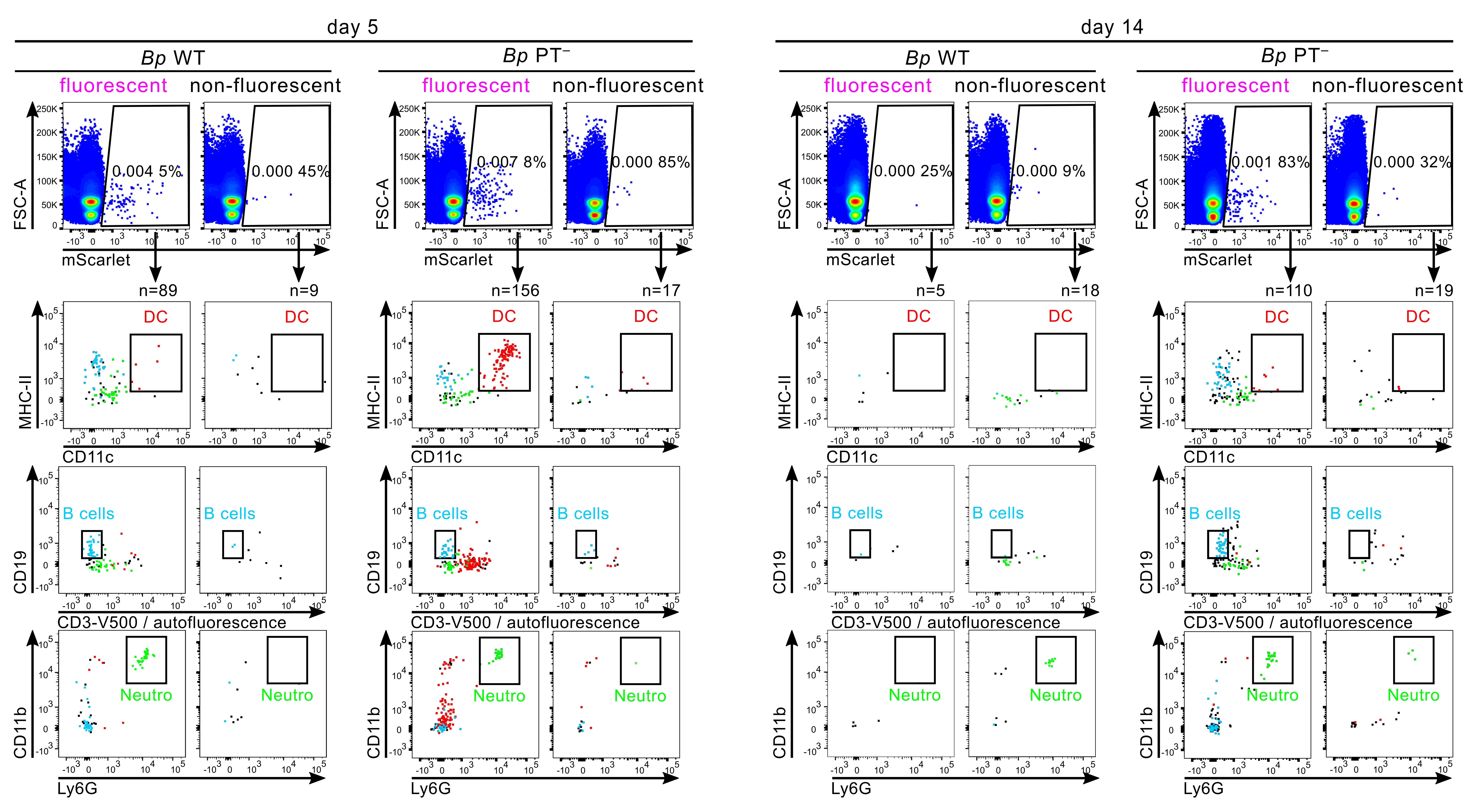

Supplement: S14 Fig — Mice were intranasally inoculated with 50 μl of bacterial suspension containing 8 x 10^5^ CFU of the indicated B. pertussis strains (mScarlet+), or with corresponding non-fluorescent strains. Cell suspensions were prepared from pools of mLNs collected on days 5 and 14 from 4 mice per each condition. mLN suspensions were pooled, stained with a panel of fluorescently-labeled antibodies and analyzed by flow cytometry. The indicated numbers represent mScarlet+ cells detected per 2 x 10^6^ events on day 5 and 14, except for day 14 of the mLNs of mice infected by Bp PT–, where the sample of analyzed cells was increased to 6 x 10^6^ events. mScarlet+ cells associated with Bp bacteria were detected using 585/15 nm emission filter and the gate was set using cellular suspension of mLNs from mice infected with the non-fluorescent control bacteria. Bp-associated cells were visualized in simple dot plots from down-sampled live mScarlet+ events (lower panels): MHC-II+ CD11c+ dendritic cells (DC, red dots), CD19+CD3– B cells (blue dots) and CD11b+Ly6G+ neutrophils (green dots). Data from one representative experiment out of 3 (Bp WT) or 4 (Bp PT–) performed are shown. (TIF) [file ppat.1010577.s016.tif]

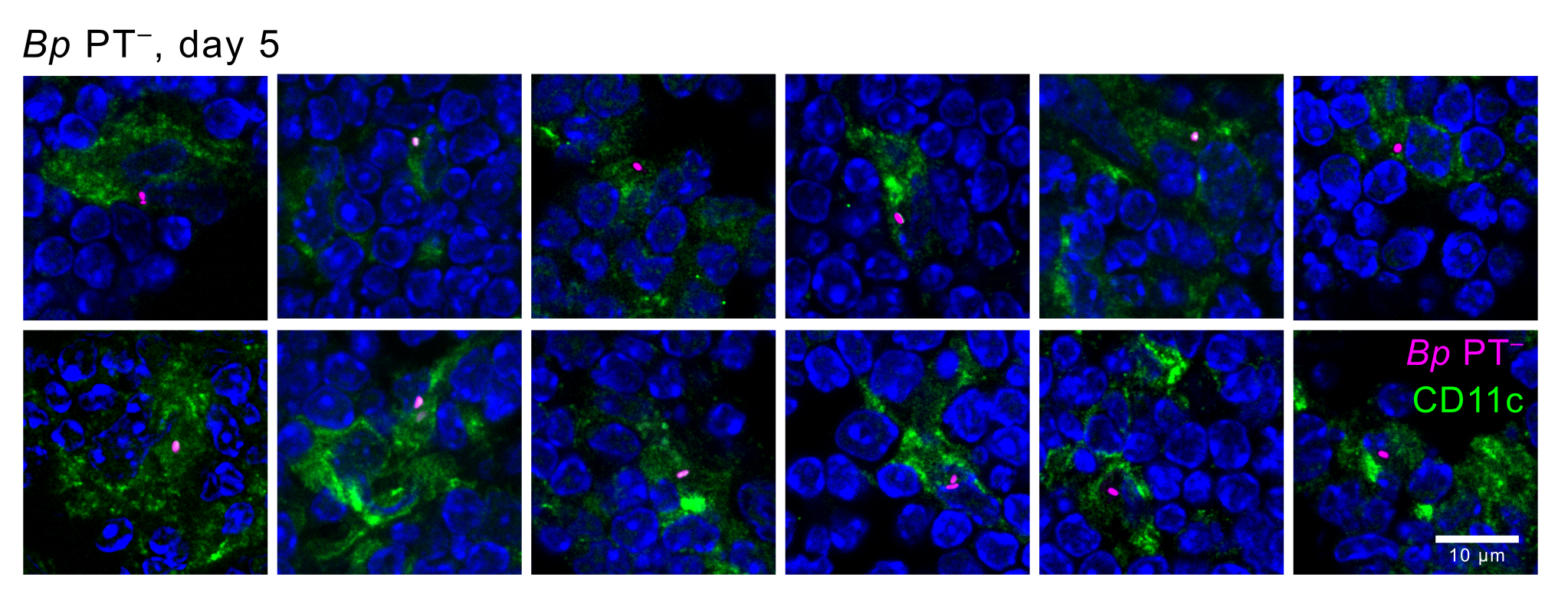

Supplement: S15 Fig — Additional images documenting the observations shown in Fig 8B. To visualize the infected dendritic cells on day 5, cryosections of mLNs from mice infected with Bp PT− mScarlet were stained by a biotin-conjugated CD11c monoclonal antibody (clone HL3), detected by AF488-conjugated streptavidin. Dendritic cells, nuclei and bacteria are rendered in green, blue, and magenta colors, respectively. Scale bar = 10 μm. (TIF) [file ppat.1010577.s017.tif]

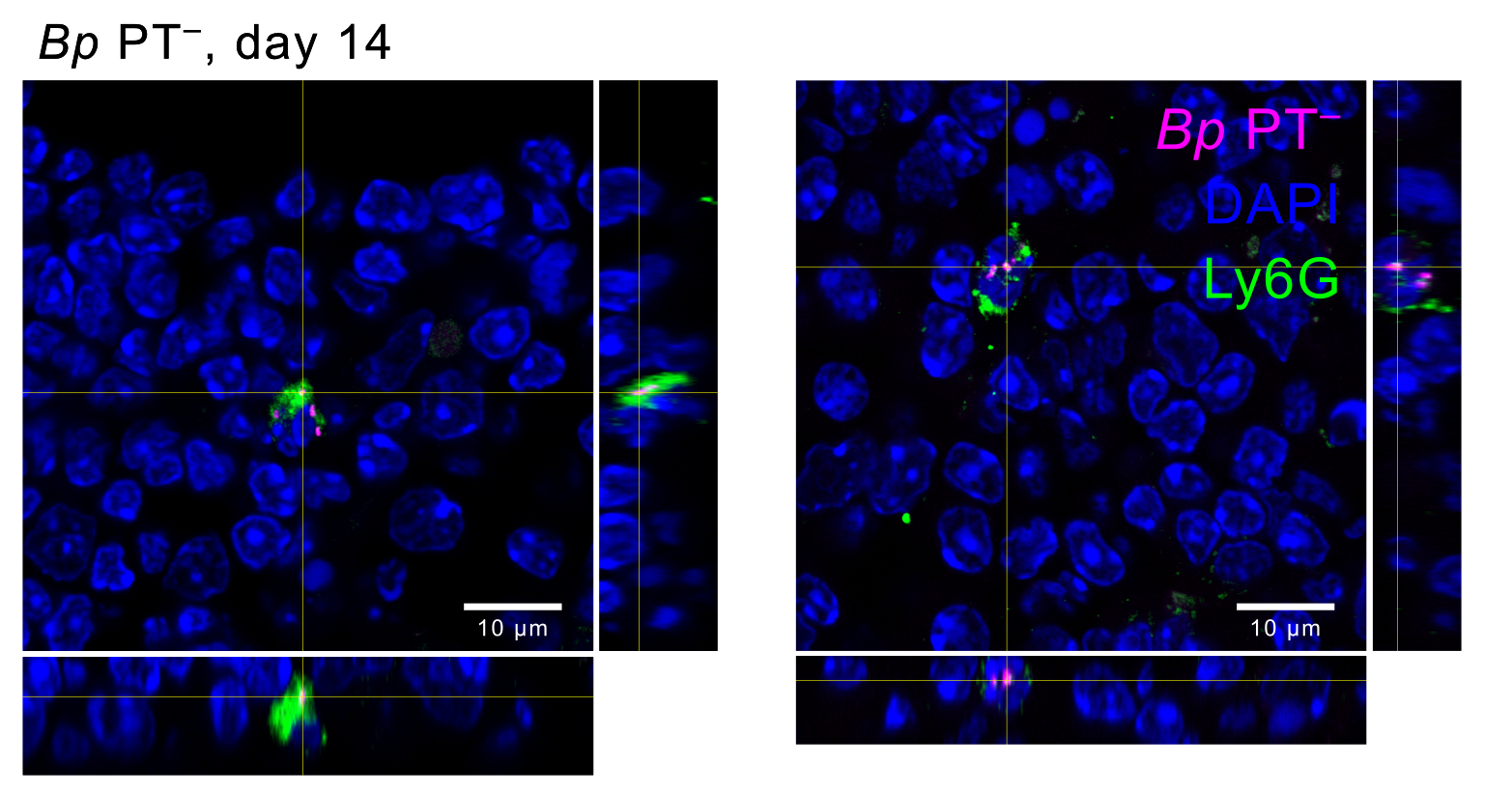

Supplement: S16 Fig — Additional images documenting the observations shown in Fig 8C. Neutrophils on cryosections of mLNs from Bp PT–-infected mice on day 14 were stained with biotin-conjugated rat Ly6G antibody followed by AF488-conjugated streptavidin. Orthogonal views of Bp PT− mScarlet-infected neutrophils clearly show intracellular localization of bacteria in neutrophils. Z-stack images were acquired using a confocal microscope. Neutrophils, nuclei and bacteria are rendered in green, blue, and magenta colors, respectively. Scale bar 10 μm. (TIF) [file ppat.1010577.s018.tif]

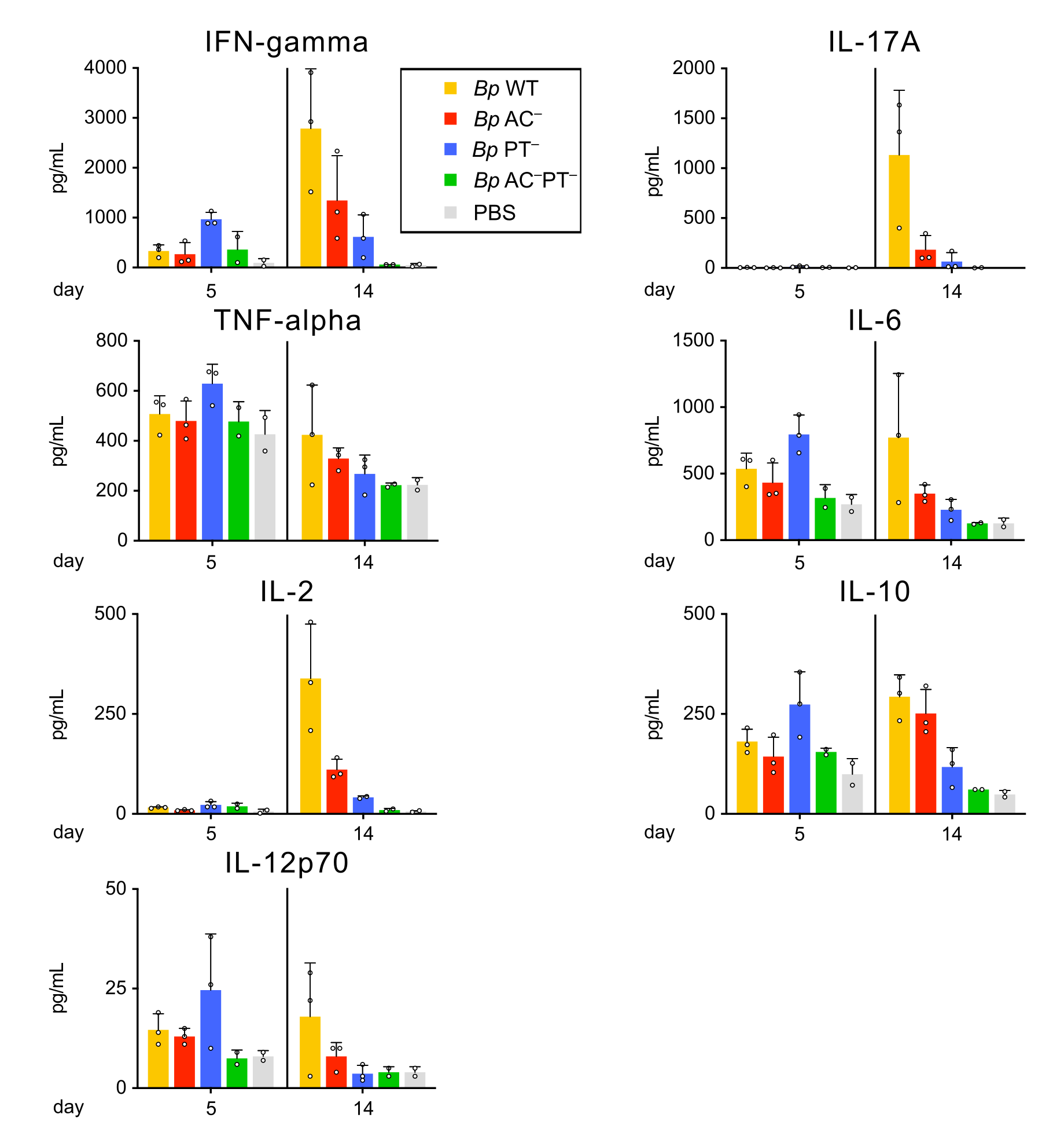

Supplement: S17 Fig — Mice were intranasally inoculated with 50 μl of bacterial suspension containing 8 x 10^5^ CFU of the indicated B. pertussis strains (mScarlet+) and on day 5 and 14 spleens were collected. Splenocytes were isolated as previously described [11] and restimulated for 48 h by heat-killed B. pertussis WT (Tohama I), or by PBS and PMA/ionomycin (eBiosciences) used as negative and positive controls, respectively. IFN-γ, IL-17A, IL-2, IL-6, IL-10, TNF-α and IL-12p70 concentrations were determined in the culture supernatants of splenocytes using a custom-made ProcartaPlex cytokine bead assay (ThermoFisher Scientific, USA) on a Bio-Plex 200 instrument (Bio-Rad, USA). The analysis was done once (n = 3 mice/group). Data are represented as means with SD. Circles represent values from individual mice. (TIF) [file ppat.1010577.s019.tif]
